# Antenatal Corticosteroids for Reducing Adverse Maternal and Child Outcomes in Special Populations of Women at Risk of Imminent Preterm Birth: A Systematic Review and Meta-Analysis

**DOI:** 10.1371/journal.pone.0147604

**Published:** 2016-02-03

**Authors:** Rachel M. Amiya, Linda B. Mlunde, Erika Ota, Toshiyuki Swa, Olufemi T. Oladapo, Rintaro Mori

**Affiliations:** 1 Department of Health Policy, National Center for Child Health and Development, Tokyo, Japan; 2 Department of Family Nursing, Graduate School of Medicine, The University of Tokyo, Tokyo, Japan; 3 Department of Community and Global Health, Graduate School of Medicine, The University of Tokyo, Tokyo, Japan; 4 Graduate School of Human Sciences, Osaka University, Suita, Japan; 5 UNDP/UNFPA/UNICEF/WHO/World Bank Special Programme of Research, Development and Research Training in Human Reproduction (HRP), Department of Reproductive Health and Research, World Health Organization, Geneva, Switzerland; Seattle Childrens Hospital, UNITED STATES

## Abstract

**Background:**

This study synthesizes available evidence on antenatal corticosteroids (ACS) use among special subgroups of women at risk of imminent preterm birth, including those (1) with pregestational and gestational diabetes mellitus, (2) undergoing elective caesarean section (CS) in late preterm (34 to<37 weeks), (3) with chorioamnionitis, and (4) with growth-restricted fetuses.

**Methods:**

A systematic search of MEDLINE, EMBASE, CINAHL, Cochrane Library, POPLINE, and World Health Organization Regional Databases was conducted for all comparative studies. Two reviewers independently determined study eligibility, extracted data, and assessed study quality. Pooled mean differences and odds ratios with 95% confidence intervals were estimated from available data, based on fixed- and random-effects models, as appropriate.

**Results:**

No eligible studies were identified for ACS use in diabetic pregnant women or those undergoing elective CS at late preterm. Nine studies each on ACS use in women with chorioamnionitis and in women with fetal growth restriction met inclusion criteria; eight studies were separately included in the meta-analyses for the two subpopulations. For ACS administration in women with chorioamnionitis, pooled analyses showed reductions in neonatal mortality (OR: 0.49, 95% CI: 0.34–0.73), respiratory distress syndrome (OR: 0.58, 95% CI: 0.44–0.76), intraventricular haemorrhage (OR: 0.41, 95% CI: 0.24–0.69), and severe intraventricular haemorrhage (OR: 0.40, 95% CI: 0.24–0.69). Maternal and long-term newborn outcomes were not reported. Effects of ACS use were inconclusive for cases with fetal growth restriction.

**Conclusion:**

Direct evidence on the effectiveness and safety of ACS is lacking for diabetic pregnant women at risk of preterm birth and those undergoing elective late-preterm CS, though this does not necessarily recommend against their use in diabetic women. While evidence remains inconclusive for women with growth-restricted preterm neonates, ACS appears to benefit preterm neonates delivered by women with chorioamnionitis. High-quality studies on maternal and long-term child outcomes in more diverse settings are needed to establish the balance of potential harms versus benefits in using ACS for these understudied subgroups.

## Background

Despite advances in medical technology, preterm birth rates have been escalating worldwide over the past two decades. Out of every ten infants born globally in 2010, it is estimated that one was preterm [1]. The resulting complications precipitated over 1 million neonatal deaths in that year alone [1]. Among children aged 5 years and younger, preterm birth complications constitute the leading cause of death [2]. Beyond being a major determinant of neonatal mortality, preterm birth has both short- and long-term consequences for the health of mother and child [3].

To alleviate such burdens, previous systematic reviews have established the effectiveness and safety of antenatal corticosteroids (ACS) for improving preterm birth outcomes in general populations of women. A Cochrane review showed that a single course of ACS significantly reduced the incidence of neonatal death by 31% [4]. Meta-analyses of morbidity data have revealed significantly reduced rates of respiratory distress syndrome (RDS), cerebroventricular haemorrhage (CVH), necrotizing enterocolitis (NEC), infectious morbidity, need for respiratory support, and neonatal intensive care unit admission with ACS treatment. For the mother, corticosteroid use does not increase the risk of death or chorioamnionitis, although a non-significant risk of puerperal sepsis was found [4].

A single course of ACS has become the standard of care in most high-income countries for cases of imminent or anticipated preterm delivery, particularly before 32 to 34 weeks’ gestation [5], representing an effective therapy for RDS and improving morbidity and mortality in preterm babies. However, key subgroups of pregnant women with potentially complicating conditions have frequently been excluded from clinical trials [4]. In particular, consensus is lacking for ACS use in women at risk of imminent preterm birth who also have pregestational and gestational diabetes; who are undergoing elective caesarean section (CS) birth in late preterm; who have evidence of intrauterine bacterial infection during labour (chorioamnionitis); and who are pregnant with growth-restricted fetuses. Recommendations from international bodies regarding the use of ACS in these important subgroups have either been omitted or based solely on expert opinions [6–8]. This lack of consistent, evidence-based guidance has continued to create significant barriers to effective clinical management of these women.

### Antenatal corticosteroids and maternal diabetes mellitus

In diabetic women, spontaneous and elective preterm births are more likely to occur compared to the general population. Preeclampsia, polyhydramnios, infections, and newborn RDS are common complications [9,10]. Infants of diabetic mothers who are born early may be prone to pulmonary immaturity at more advanced stages of gestation than infants of non-diabetic mothers [9,11]. The need for ACS therapy is thus likely to be greater in the presence of pregestational or gestational diabetes mellitus. Indeed, an independent effect of maternal diabetes mellitus on the risk of severe respiratory complications has been demonstrated in both term and preterm infants [12,13]. Yet, importantly, such subgroups of women have been excluded from most randomized controlled trials (RCTs) of ACS therapy due to concerns about the potential effects on glycaemic control [4]. As the prevalence of gestational diabetes mellitus increases, so too does the urgency of tackling questions about indications for ACS use in the context of pre-existing, overt, or gestational diabetes mellitus complicated by threatened preterm birth.

### Antenatal corticosteroids and late-preterm elective caesarean birth

Evidence indicates that infants born by CS, both at term and preterm, are more susceptible to respiratory morbidity—primarily RDS and transient tachypnoea—than infants born vaginally [14,15], and this risk increases further for babies born after *elective* CS (i.e., CS before onset of labour or prelabour CS) [16,17]. As with pregnancies complicated by maternal diabetes, women who have elective CS may require ACS therapy. However, while evidence clearly supports routine ACS use when birth is expected before 34 weeks’ gestation [7], specific guidance on ACS use in mothers undergoing elective CS in *late* preterm (34–36 weeks + 6 days) is lacking.

### Antenatal corticosteroids and maternal intrapartum bacterial infection

There is debate and general concern regarding the safety and efficacy of ACS for preterm birth in cases of suspected intrauterine infection [6,18]. Given their immunosuppressive effects, corticosteroids could theoretically activate latent infections or worsen fungal infections, leading many to raise concerns over the potential risk to mother and baby. Accordingly, chorioamnionitis is frequently cited as a contraindication for ACS therapy [6,8,19] and its clinical signs often used as an exclusion criterion for related clinical trials [4,18,20]. This has led to a critical lack of data regarding the effects of ACS on neonatal outcomes in the context of suspected maternal intrapartum bacterial infection [21] and, therefore, an uncertain scientific basis for guidelines recommending against their use in such cases. In fact, despite widespread reservations surrounding the use of steroids in pregnancies complicated by chorioamnionitis, the positive findings of single studies [18,22,23] and one previous systematic review [24] suggest that a more liberal application of ACS in such cases may be warranted. Given the rapid pace of development in this field and continuing controversy surrounding the subject, an updated systematic review becomes necessary.

### Antenatal corticosteroids and fetal growth restriction

Some researchers have postulated that the chronic intrauterine stress associated with fetal growth restriction may stimulate the fetal adrenal gland to produce cortisol, leading to enhanced fetal lung maturation [25,26]. In addition, due to breakdown of the enzyme responsible for blocking passage of maternal cortisol across the placenta, growth-restricted fetuses are exposed to higher levels of maternal steroids [27]. Under such conditions, exogenous administration of corticosteroids may be expected to bring no added benefit in growth-restricted fetuses. Indeed, preterm growth-restricted fetuses have exhibited divergent cardiovascular responses to ACS treatment in human blood flow studies, with one study finding no observable effect on fetal Doppler waveform patterns [28] and two other studies showing altered resistance to fetoplacental blood flow [29,30].

Aside from the lack of consistent evidence of advantageous outcomes, early applications of ACS in mothers with severe hypertension and in those with growth-restricted fetuses have also been linked to adverse fetal effects [31]. Indeed, exposure to steroids, particularly repeat doses, during pregnancy has itself been associated with reduced fetal growth [32]. In growth-restricted animal models, meanwhile, ACS therapy has been shown to alter cerebral blood flow, impair brain growth, and cause brain damage in the developing fetus [32]. Moreover, both antenatal steroid exposure and growth restriction itself have been hypothesized as key mechanisms underlying the fetal origins of adult disease hypothesis [33]. Based on these concerns, mothers of growth-restricted fetuses, as with the aforementioned subgroups, have largely been excluded from large RCTs of ACS therapy [4,20]. Again, this creates serious limitations for informed decision making, and direct evidence is thus critically needed to guide practice in cases of mothers with growth-restricted fetuses at imminent risk of preterm birth.

### Objectives

The highlighted gaps in the evidence base demand an examination of the implications of using or not using ACS in cases of imminent preterm birth in these subgroups of women. As part of efforts to complement the evidence base preparation for the *World Health Organization (WHO) recommendations on interventions to improve preterm birth outcomes* [34], we performed a systematic review to assess the effects on maternal and child outcomes of ACS administration in four less common but equally important populations of pregnant women at risk of imminent preterm birth. These subgroups were as follows:

1. women with pregestational and gestational diabetes mellitus;
2. women undergoing elective CS in late preterm;
3. women with intrapartum bacterial infections; and
4. women with growth-restricted fetuses.

## Methods

### Study eligibility criteria

Eligible studies included all published, unpublished, and ongoing randomized or quasi-randomized controlled trials, controlled before-and-after studies, interrupted-time-series studies, historically controlled studies, cohort studies, and cross-sectional studies comparing ACS administration (betamethasone, dexamethasone, or hydrocortisone), given either parenterally or enterally, compared with placebo or no treatment in women at risk of imminent preterm birth as a result of either spontaneous preterm labour, preterm prelabour rupture of the membranes, or elective preterm delivery, and where all (or at least a well-defined sub-sample) of the women under study also fulfilled one or more of the following conditions:

1. having pregestational or gestational diabetes mellitus (*sub-question P1*);
2. undergoing elective caesarean birth in late preterm (34 weeks to <36 weeks + 6 days) (*sub-question P2*);
3. having an intrapartum bacterial infection (e.g., chorioamnionitis, systemic infections) (*sub-question P3*); or
4. having a growth-restricted infant (or, more broadly, one that was at least small for gestational age [SGA]^a^; *sub-question P4*).

Articles in any language and from any country were eligible for inclusion if they reported one or more neonatal outcome measure of interest regarding antenatal steroid status in preterm infants delivered by mothers fitting the aforementioned inclusion criteria. The following were maternal outcomes of interest for the review: death or severe morbidity (e.g., organ dysfunction, intensive care unit admission, chorioamnionitis, pregnancy-induced hypertension, gestational diabetes mellitus, placental abruption, postpartum haemorrhage, or as defined by the author), route of delivery, and side effects of therapy. For the newborn and child outcomes, the following were outcomes of interest for the review: neonatal mortality, perinatal mortality, RDS, surfactant use, intraventricular haemorrhage (IVH), periventricular leukomalacia (PVL), neonatal sepsis, NEC, mechanical ventilation use and duration, patent ductus arteriosus (PDA), chronic lung disease (CLD), bronchoplumonary dysplasia (BPD), Apgar scores, low birth weight, neurodevelopment, and anthropomentric status.

In terms of comparisons, we considered all studies that incorporated a placebo or suitable control group comparable to the experimental group aside from not receiving ACS. ACS could be administered either alone or in combination with antibiotics and surfactants.

### Data sources and search strategy

An information specialist with expertise in conducting systematic reviews developed the search strategy in collaboration with study authors. Systematic searches of electronic databases including MEDLINE, EMBASE, CINAHL, Cochrane Library, POPLINE, and all World Health Organization Regional Databases were undertaken on February 13 and 14, 2014, with no date restrictions. Controlled vocabulary, supplemented with free keywords, was used to search for the relevant concept areas, with duplicates removed in the process to yield a total number of abstracts for each database (see S1 File for detailed database-specific search strategies and terms).

Reference lists of obtained articles, including any recent systematic reviews, were also hand searched for further potentially relevant studies. Additionally, the Cochrane Register of Controlled Trials (CENTRAL), ClinicalTrials.gov, International Standard Randomised Controlled Trial Number Register (ISRCTN), and the International Clinical Trials Registry Platform (ICTRP) were searched using key terms to identify any relevant unpublished materials. All citations were imported into an EndNote (EndNote X5, Thomson Reuters, New York, NY, USA) library for processing.

### Study selection and data extraction

Two reviewers (RMA, LBM) worked independently to assess each title and abstract for eligibility. Disagreements yielded automatic inclusion into the next level of screening. After initial screening of titles and abstracts, full-text publications of studies with the potential for inclusion were obtained and assessed. The same two reviewers independently evaluated studies under consideration for inclusion without consideration of their results. Any disagreements were resolved by discussion to reach consensus. Finally, the two reviewers independently extracted baseline and outcome data and assessed the quality of the included studies. Any discrepancies were resolved through discussion to reach a consensus. Data were entered into Review Manager version 5 software (RevMan 5.3; The Cochrane Collaboration, Oxford, UK) and checked for accuracy. All outcomes were assessed as individually defined by the original study investigators.

### Assessment of study quality and publication bias

Study quality was assessed independently by the two aforementioned reviewers at the outcome level using the Newcastle-Ottawa Scale (NOS) [35] for cohort studies as well as the Risk of Bias Assessment tool for Non-randomized Studies (RoBANS) [36], out of which comparable assessments were achieved. Potential publication bias was assessed by visual inspection of funnel plots for asymmetry, subject to a sufficient number of included studies [37]. Again, any disagreement was resolved by consensus.

### Data synthesis and analysis

Aggregate odds ratios (ORs) and 95% confidence intervals (CIs) were calculated for dichotomous data using Mantel-Haenszel analysis (fixed-effects model). Where between-study clinical or methodological heterogeneity undermined the compatibility of the quantitative results, or if substantial statistical heterogeneity was detected, random-effects meta-analysis was used. Data were pooled using ORs when the numbers of events was available, and using logarithms of the ORs weighted by the inverse variance when events were not available. For continuous data, mean differences (MDs) with 95% CIs were used.

The heterogeneity of studies was assessed using both qualitative and quantitative measures. Statistical heterogeneity was determined for each meta-analysis using T^2^, I^2^, and Chi^2^ statistics. Heterogeneity was deemed substantial if T^2^ was greater than zero and either I^2^ was greater than 50% or p < 0.10 in the Chi^2^ test for heterogeneity [38]. To further assess potential heterogeneity effects, both fixed- and random-effects models were compared for each outcome, where possible.

All statistical analyses were performed using RevMan 5. Existing meta-analyses were reviewed for relevance and completeness, and new meta-analyses were performed where deemed necessary. Statistical significance was set at an alpha level of 0.05 for all analyses, except when testing study heterogeneity, where p < 0.10 was regarded as significant. Evidence profiles were prepared for each research question using the Web-based Guideline Development Tool (GDT; www.guidelinedevelopment.org).

## Results

### Results for sub-question P1: pregestational and gestational diabetic women

**Fig 1:**
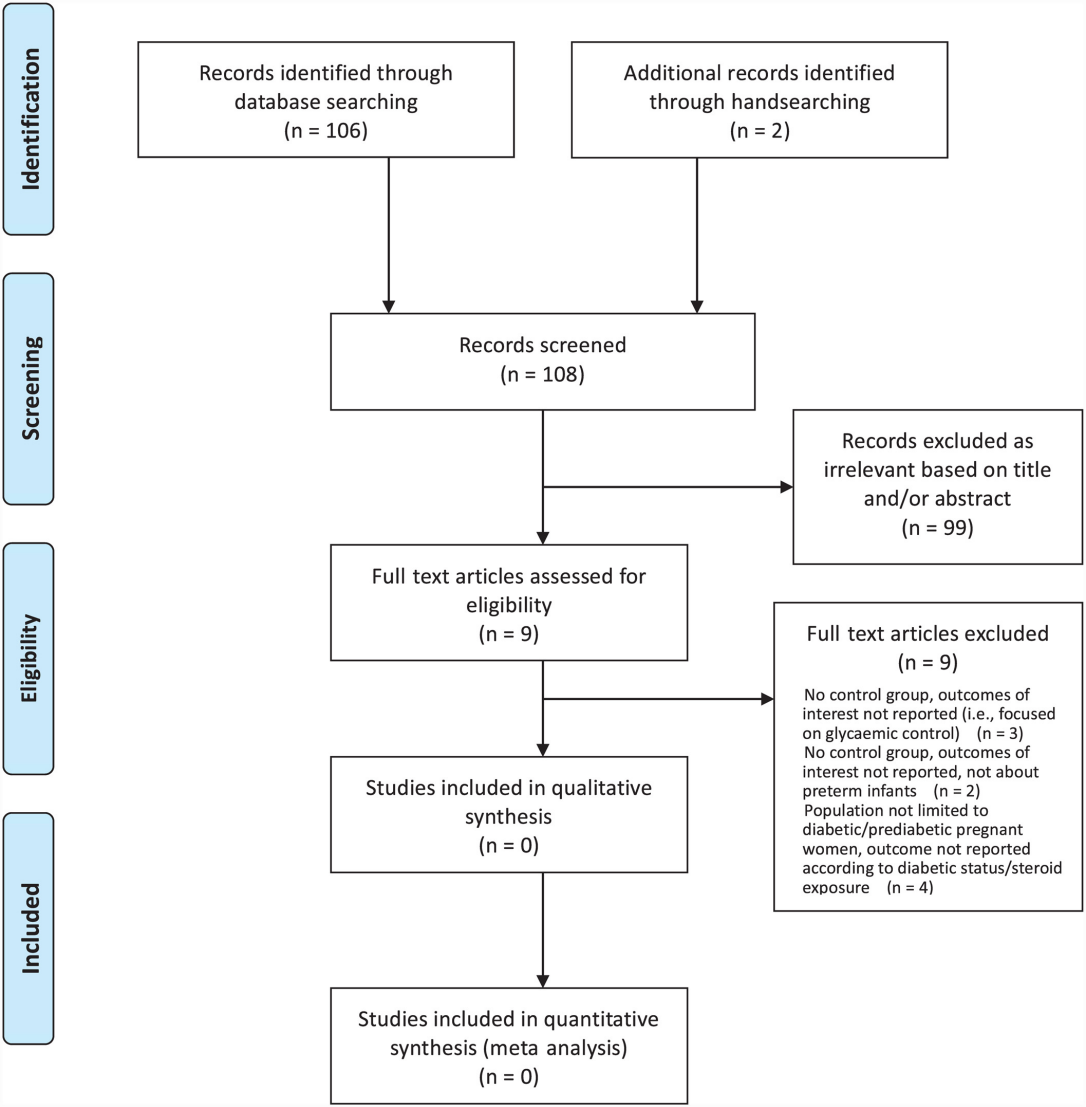
Flow diagram of search results and study selection for sub-question P1.

Our search identified 106 citations from the electronic databases and 2 further articles by reviewing reference lists of relevant primary research and review articles for sub-question P1. After title and abstract review, we obtained the full texts of nine articles, out of which no studies met the inclusion criteria (Fig 1).

### Results for sub-question P2: women undergoing elective CS in late preterm

**Fig 2:**
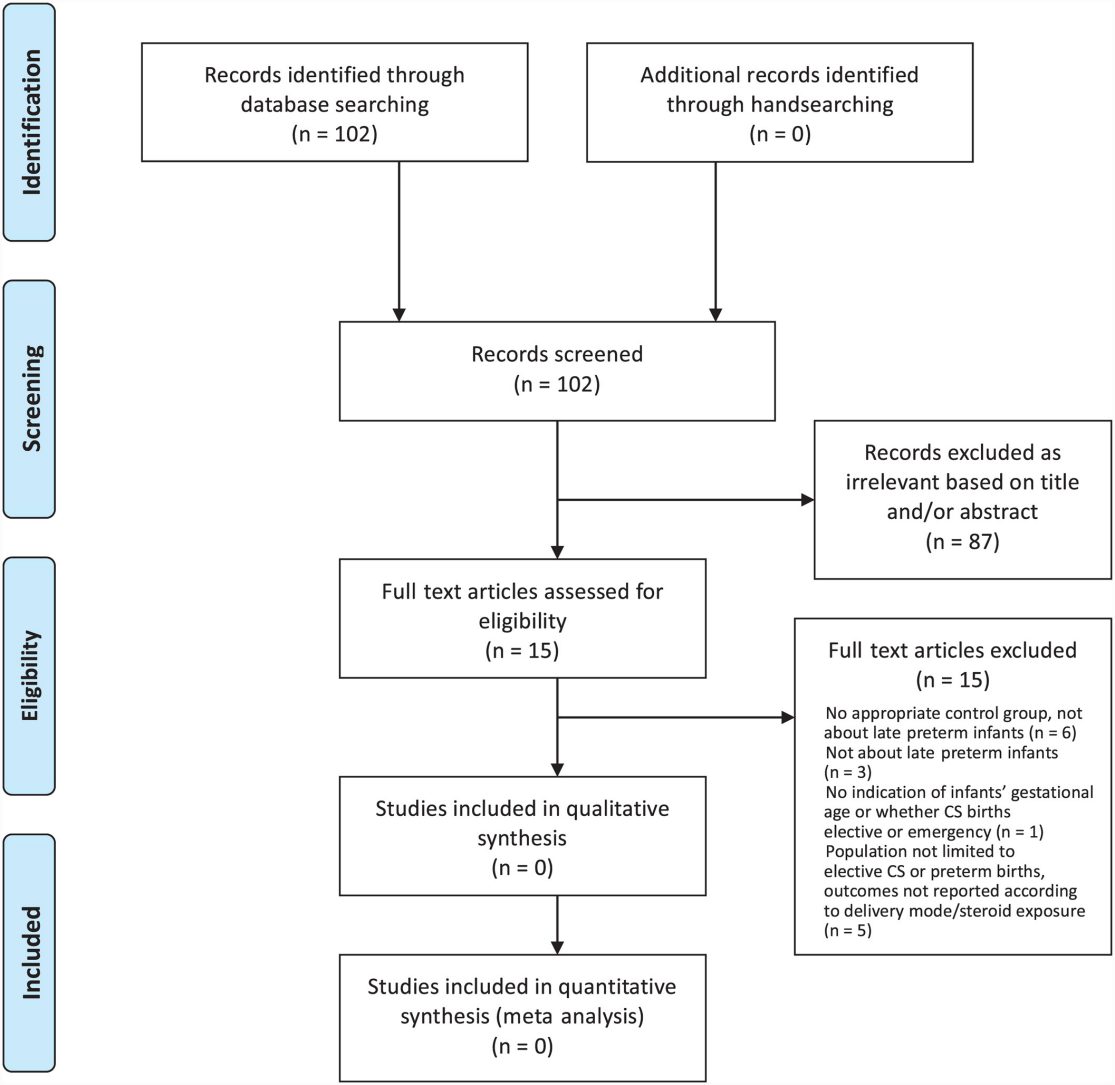
Flow diagram of search results and study selection for sub-question P2.

Our search identified 102 citations for sub-question P2. Following title and abstract review, full texts of 15 articles were obtained, out of which no studies met the inclusion criteria (Fig 2).

### Results for sub-question P3: women with chorioamnionitis (histological or clinical)

**Fig 3:**
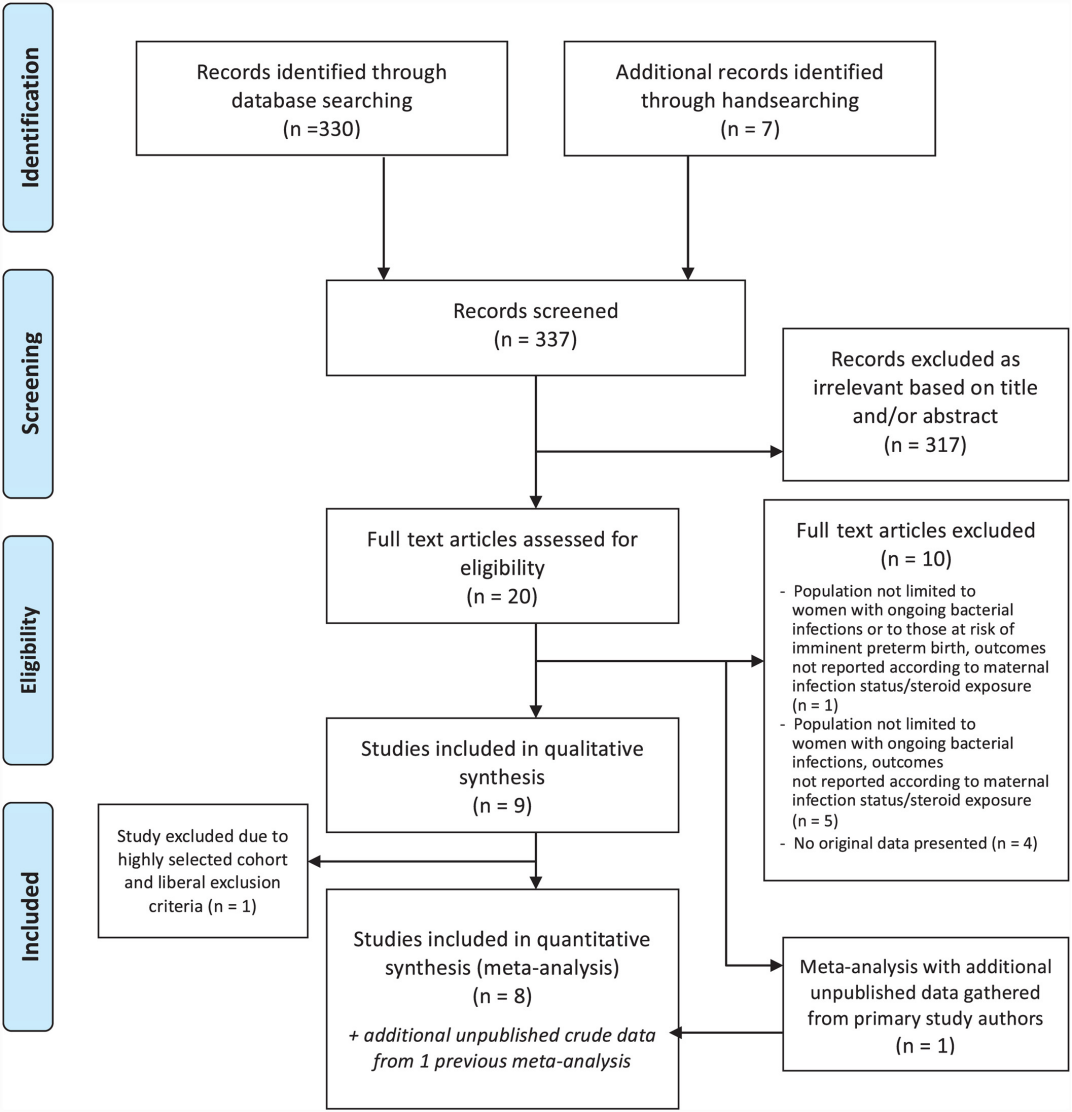
Flow diagram of search results and study selection for sub-question P3.

Our search identified 330 candidate citations from the electronic databases and 7 further articles by reviewing reference lists of relevant primary research and review articles for sub-question P3. Full texts were obtained for 20 articles based on title and abstract review. After excluding ineligible studies, nine studies met the inclusion criteria, out of which eight studies could be synthesized quantitatively (Fig 3), including data from a total of 1424 mother/newborn dyads [21,22,39–44]. One small study [45] was excluded from the meta-analyses because its highly selected cohort and liberal exclusion criteria complicated extrapolation of the findings. The general characteristics of the eight studies included in the meta-analysis are summarized in Table 1. Additional unpublished crude data from five [21,22,40,43,44] of these eight primary studies were further extracted from a previous meta-analysis [24] identified through the search process. Inclusion criteria for all eight studies was gestational age below 34 weeks, and less than 32 weeks in six out of the eight studies. Furthermore, all studies were conducted in high-income countries with advanced maternal and newborn care including NICU.

**Table 1:** Basic characteristics of the included studies for sub-question P3 (women with chorioamnionitis). HC, histological chorioamnionitis; CC, clinical chorioamnionitis; Dexa, Dexamethasone; Beta, Betamethasone; RDS, respiratory distress syndrome; BPD, bronchopulmonary dysplasia; IHC, intrahepatic cholestasis; IVH, intraventricular haemorrhage; PVL, periventricular leukomalacia; NEC, necrotizing enterocolitis; PDA, patent ductus arteriosus; CLD, chronic lung disease.

Author, year	Study design	N (treatment, control)	Enrollment period	Location	Inclusion criteria	Exclusion criteria	HC or CC?	Antenatal corticosteroid course					Outcomes reported
								Drug	Dose (mg)	Interval (hrs)	ACS cut-off ^a^	Repeat?	
Ahn et al., 2013 [38]	Prospective cohort	89 (52, 36)	2005–2010	South Korea	<34 wks	Major congenital anomalies; outborn	HC	Dexa	5	12	None ^b^	Not reported	RDS; Mechanical ventilation; Duration of ventilation; BPD; Sepsis; IHC; PVL; NEC, PDA; Mortality
Baud et al., 2000[39]	Retrospective cohort	170 (60, 110)	1990–1997	France	<33 wks singletons	Severe diabetes mellitus; multiple malformations	CC	Beta/Dexa	12/6	12/24	≥1x	Yes	RDS; BPD; CLD; Cystic PVL; Hemodynamic failure; Neonatal sepsis; Severe IVH; Mortality
Been et al., 2009 [21]	Prospective cohort	121 (89, 32)	2001–2003	Netherlands	<32 wks	Congenital anomalies	HC, CC	Beta	12	24	≥2x	No	RDS; BPD; IVH; Severe IVH; PVL; NEC; PDA; Sepsis; Mortality
Dempsey et al., 2005 [40]	Retrospective cohort	130 (88, 42)	1989–1999	USA	<30 wks singletons	None	HC	Beta	12	24	≥2x	Not reported	RDS; IVH; NEC; Sepsis; Pneumonia; Meningitis; Mortality
Elimian et al., 2000[41]	Retrospective cohort	527 (169, 358)	1990–1997	USA	500–1750g	CC	HC	Beta	12	24	≥2x	Yes	RDS; NEC; PDA; Sepsis; 5-min Apgar score<7; Surfactant; IVH/PVL; Major brain lesions; Mortality
Foix-L’Helias et al., 2005[42]	Retrospective cohort	97 (45, 52)	1993–1996	France	24–31 wks singleton	None	CC	Beta/Dexa	12/6	12/24	≥1x	Yes	RDS; BPD; Mortality
Goldenberg et al., 2006 [22]	Retrospective cohort	218 (182, 36)	1996–2001	USA	23–32 wks singletons	None	HC, CC	Beta	12	24	≥1x	Yes ^c^	RDS; BPD; Severe IVH; PVL; NEC; Sepsis; Mortality
Kent et al., 2005 [43]	Prospective cohort	72 (58, 14)	1996–2001	Australia	<30 wks	None	HC	Dexa	12	12	≥2x	Yes	IVH; Severe IVH; Mortality

^a^ Refers to the number of antenatal steroid doses used as a cut-off to determine antenatal corticosteroid administration as positive.

^b^ The study included infants who received incomplete courses of antenatal corticosteroids.

^c^ Rescue course in selected cases ≥1 week after initial full course.

Table 2 summarizes the findings of the studies and the quality of the evidence for ACS use in women with chorioamnionitis, with pooled results shown separately for histologically and clinically diagnosed chorioamnionitis (see S2 File for Forest plots). Applying a random-effects model did not yield substantially different effect sizes or significance levels for any outcome measure [42]. None of the studies included in the review reported on any maternal outcomes, only one study followed up through childhood, and none followed up through adulthood. No significantly elevated risk was detected for any adverse outcome following ACS therapy.

**Table 2:** GRADE evidence profile for sub-question P3 (women with chorioamnionitis). HC, histological chorioamnionitis; CC, clinical chorioamnionitis; CI, confidence interval; OR, odds ratio; MD, mean difference.

Quality assessment							No. of patients		Effect		Quality
No. of studies	Study design	Risk of bias	Inconsistency	Indirectness	Imprecision	Other considerations	Antenatal corticosteroids	No antenatal corticosteroids	Relative (95% CI)	Absolute (95% CI)	
**Neonatal death (HC)**											
6	observational studies	serious ^a^	not serious	not serious	serious ^b^	not serious	64/638 (10.0)%	89/518 (17.2)%	OR 0.49 (0.34 to 0.73)	80 fewer per 1000 (from 40 fewer to 106 fewer)	⊕○○○ VERY LOW
**Neonatal death (CC)**											
3	observational studies	serious ^a^	not serious	not serious	very serious ^c^	not serious	17/149 (11.4)%	15/98 (15.3)%	OR 0.77 (0.36 to 1.65)	31 fewer per 1000 (from 77 more to 92 fewer)	⊕○○○ VERY LOW
**Respiratory distress syndrome (HC)**											
5	observational studies	serious ^a^	not serious	not serious	serious ^b^	not serious	279/580 (48.1)%	285/504 (56.5)%	OR 0.58 (0.44 to 0.76)	135 fewer per 1000 (from 68 fewer to 201 fewer)	⊕○○○ VERY LOW
**Respiratory distress syndrome (CC)** ^d^											
4	observational studies	not serious	not serious	not serious	serious ^b^	not serious	99/209 (47.4)%	99/208 (47.6)%	OR 0.73 (0.48 to 1.12)	77 fewer per 1000 (from 28 more to 172 fewer)	⊕○○○ VERY LOW
**Surfactant use (HC)**											
3	observational studies	serious ^a^	not serious	not serious	serious ^b^	not serious	187/316 (59.2)%	244/404 (60.4)%	OR 0.93 (0.67 to 1.3)	17 fewer per 1000 (from 61 more to 99 fewer)	⊕○○○ VERY LOW
**Intraventricular haemorrhage (HC)**											
5	observational studies	not serious	not serious	not serious	serious ^b^	strong association	53/463 (11.4)%	32/158 (20.3)%	OR 0.41 (0.24 to 0.69)	108 fewer per 1000 (from 53 fewer to 145 fewer)	⊕⊕○○ LOW
**Intraventricular haemorrhage (CC)**											
3	observational studies	not serious	not serious	not serious	serious ^b^	strong association	13/163 (8.0)%	20/155 (12.9)%	OR 0.36 (0.16 to 0.82)	78 fewer per 1000(from 21 fewer to 106 fewer)	⊕⊕○○ LOW
**Severe intraventricular haemorrhage (HC)**											
4	observational studies	not serious	not serious	not serious	serious ^b^	strong association	28/375 (7.5)%	16/116 (13.8)%	OR 0.40 (0.20 to 0.79)	78 fewer per 1000 (from 26 fewer to 107 fewer)	⊕⊕○○ LOW
**Severe intraventricular haemorrhage (CC)**											
3	observational studies	not serious	not serious	not serious	very serious ^c^	strong association	5/163 (3.1)%	14/155 (9.0)%	OR 0.29 (0.10 to 0.89)	62 fewer per 1000(from 9 fewer to 80 fewer)	⊕⊕○○ VERY LOW
**Periventricular leukomalacia (HC)**											
3	observational studies	not serious	not serious	not serious	very serious ^c^	not serious	13/317 (4.1)%	6/102 (5.9)%	OR 0.74 (0.26 to 2.09)	15 fewer per 1000 (from 43 fewer to 57 more)	⊕○○○ VERY LOW
**Periventricular leukomalacia (CC)**											
3	observational studies	not serious	not serious	not serious	serious ^b^	strong association	8/163 (4.9)%	24/155 (15.5)%	OR 0.35 (0.14 to 0.85)	95 fewer per 1000 (from 20 fewer to 130 fewer)	⊕○○○ VERY LOW
**Neonatal sepsis (HC)**											
5	observational studies	not serious	not serious	not serious	serious ^b^	not serious	87/580 (15.0)%	80/504 (15.9)%	OR 1.03 (0.72 to 1.48)	4 more per 1000 (from 39 fewer to 60 more)	⊕○○○ VERY LOW
**Neonatal sepsis (CC)**											
2	observational studies	not serious	not serious	not serious	very serious ^c^	not serious	26/104 (25.0)%	12/46 (26.1)%	OR 0.94 (0.40 to 2.18)	12 fewer per 1000 (from 137 fewer to 174 more)	⊕○○○ VERY LOW
**Necrotizing enterocolitis (HC)**											
5	observational studies	serious ^a^	not serious	not serious	serious ^b^	not serious	60/580 (10.3)%	30/504 (5.9)%	OR 1.33 (0.78 to 2.26)	18 more per 1000 (from 12 fewer to 66 more)	⊕○○○ VERY LOW
**Necrotizing enterocolitis (CC)**											
2	observational studies	serious ^a^	not serious	not serious	very serious ^c^	not serious	16/104 (15.4)%	3/46 (6.5)%	OR 2.63 (0.72 to 9.68)	90 more per 1000 (from 17 fewer to 338 more)	⊕○○○ VERY LOW
**Duration of mechanical ventilation, days (HC)**											
1	observational studies	not serious	not serious	not serious	very serious ^c^	not serious	52	36	-	MD 2 lower (4.23 lower to 0.23 higher)	⊕○○○ VERY LOW
**Use of mechanical ventilation (HC)**											
1	observational studies	not serious	not serious	not serious	very serious ^c^	not serious	66/89 (74.2)%	29/32 (90.6)%	OR 0.30 (0.08 to 1.07)	163 fewer per 1000 (from 6 more to 470 fewer)	⊕○○○ VERY LOW
**Use of mechanical ventilation (CC only)**											
1	observational studies	not serious	not serious	not serious	serious ^e^	not serious	49/64 (76.6)%	29/29 (100)%	OR 0.05 (0.00 to 0.94)	0 fewer per 1000 (from 0 fewer to 0 fewer)	⊕○○○ VERY LOW
**Chronic lung disease / Bronchopulmonary dysplasia (HC)**											
3	observational studies	not serious	not serious	not serious	serious ^b^	not serious	55/323 (17.0)%	26/104 (25.0)%	OR 0.66 (0.38 to 1.14)	83 fewer per 1000 (from 25 more to 138 fewer)	⊕○○○ VERY LOW
**Chronic lung disease / Bronchopulmonary dysplasia (CC)**											
3	observational studies	serious ^a^	not serious	not serious	very serious ^c^	not serious	25/142 (17.6)%	16/90 (17.8)%	OR 0.91 (0.44 to 1.86)	13 fewer per 1000 (from 91 fewer to 109 more)	⊕○○○ VERY LOW
**5-minute Apgar score <7 (HC)**											
1	observational studies	serious ^a^	not serious	not serious	very serious ^c^	strong association	31/169 (18.3)%	120/358 (33.5)%	OR 0.45 (0.28 to 0.7)	150 fewer per 1000 (from 74 fewer to 211 fewer)	⊕○○○ VERY LOW
**Cerebral palsy (at 1 and 3 years' follow-up) (HC)**											
1	observational studies	serious ^a^	not serious	not serious	very serious ^c^	not serious	5/58 (8.6)%	3/14 (21.4)%	OR 0.35 (0.07 to 1.67)	127 fewer per 1000 (from 99 more to 196 fewer)	⊕○○○ VERY LOW
**General Development Quotient at 1 years' follow-up (HC)**											
1	observational studies	serious ^a^	not serious	not serious	very serious ^c^	not serious	58	14	-	MD 6 higher (8.94 lower to 20.94 higher)	⊕○○○ VERY LOW
**General Development Quotient at 3 years' follow-up (HC)**											
1	observational studies	serious ^a^	not serious	not serious	very serious ^c^	not serious	58	14	-	MD 13 higher (3.75 lower to 29.75 higher)	⊕○○○ VERY LOW

^a^ Evidence heavily based on studies with design limitations including lack of adjustment for potential confounding factors.

^b^ Estimate based on wide confidence interval crossing the line of no effect.

^c^ Estimate based on small sample size; wide confidence interval crossing the line of no effect.

^d^ A 10% cohort overlap between the studies by Foix-L’Helias et al. and Baud et al. has been reported,[24] which was not accounted for in the meta-analysis. This may have slightly affected the pooled estimate on RDS after clinical chorioamnionitis.

^e^ Estimate based on small sample size.

Administration of ACS for women with histological chorioamnionitis was associated with significant reductions in neonatal death (pooled OR: 0.49, 95% CI: 0.34–0.73; 6 studies, 1156 infants), RDS (pooled OR: 0.58, 95% CI: 0.44–0.76; 5 studies, 1084 infants), IVH (all) (pooled OR: 0.41, 95% CI: 0.24–0.69; 5 studies, 621 infants), and severe IVH (grade 3–4) (pooled OR: 0.40, 95% CI: 0.20–0.79; 4 studies, 491 infants). One study found a significant reduction in the incidence of infants with Apgar score < 7 associated with ACS therapy (OR: 0.45, 95% CI: 0.28–0.70; 527 infants). In another study, meanwhile, no significant differences between exposed and control groups were observed in the need for mechanical ventilation use (OR: 0.30, 95% CI: 0.08–1.07; 121 infants), nor in the duration of mechanical ventilation (MD: -2.00, 95% CI: -4.23–0.23; 88 infants). Likewise, no significant differences were observed in PVL (pooled OR: 0.74, 95% CI: 0.26–2.09; 3 studies, 419 infants), neonatal sepsis (pooled OR: 1.03, 95% CI: 0.72–1.48; 5 studies, 1084 infants), NEC (pooled OR: 1.33, 95% CI: 0.78–2.26; 5 studies, 1084 infants), surfactant use (pooled OR: 0.93, 95% CI: 0.67–1.30; 3 studies, 720 infants), or CLD/BPD (pooled OR: 0.66, 95% CI: 0.38–1.14; 3 studies, 427 infants). One small study that followed up through childhood was unable to show any difference in incidence of cerebral palsy (OR: 0.35, 95% CI: 0.07–1.67; 72 children) or neurodevelopmental outcome (general development quotient) at the ages of 1 year (MD: 6.00, 95% CI: -8.94–20.94; 72 children) or 3 years (MD: 13.00, 95% CI: -3.75–29.75; 72 children).

In women with clinical chorioamnionitis, ACS therapy was not associated with significant differences in neonatal mortality (pooled OR: 0.77, 95% CI: 0.36–1.65; 3 studies, 247 infants), RDS (pooled OR: 0.73, 95% CI: 0.48–1.12; 4 studies, 417 infants), neonatal sepsis (pooled OR: 0.94, 95% CI: 0.40–2.18; 2 studies, 150 infants), or NEC (pooled OR: 2.63, 95% CI: 0.72–9.68; 2 studies, 150 infants). Significant reductions were, however, observed in incidence of IVH (pooled OR: 0.36, 95% CI: 0.16–0.82; 3 studies, 318 infants), severe IVH (pooled OR: 0.29, 95% CI: 0.10–0.89; 3 studies, 318 infants) and PVL (pooled OR: 0.35, 95% CI: 0.14–0.85; 3 studies, 318 infants) among infants of mothers treated with ACS. In one study, corticosteroid therapy also significantly decreased the need for mechanical ventilation (OR: 0.05, 95% CI: 0.00–0.94; 93 infants). No significant differences were observed in the frequencies of CLD/BPD (pooled OR: 0.91, 95% CI: 0.44–1.86; 3 studies, 232 infants).

Quality of the evidence was graded as very low across all measured outcomes except IVH and severe IVH, for which the evidence quality was graded as low. Visual indication of some degree of publication bias was evident only from the funnel plot for the data on perinatal death and on major brain lesion as outcomes of ACS therapy in women with SGA infants. See S3 File for a full summary of the risk of bias assessment undertaken for each of the included studies.

### Results for sub-question P4: women with SGA/growth-restricted infants

**Fig 4:**
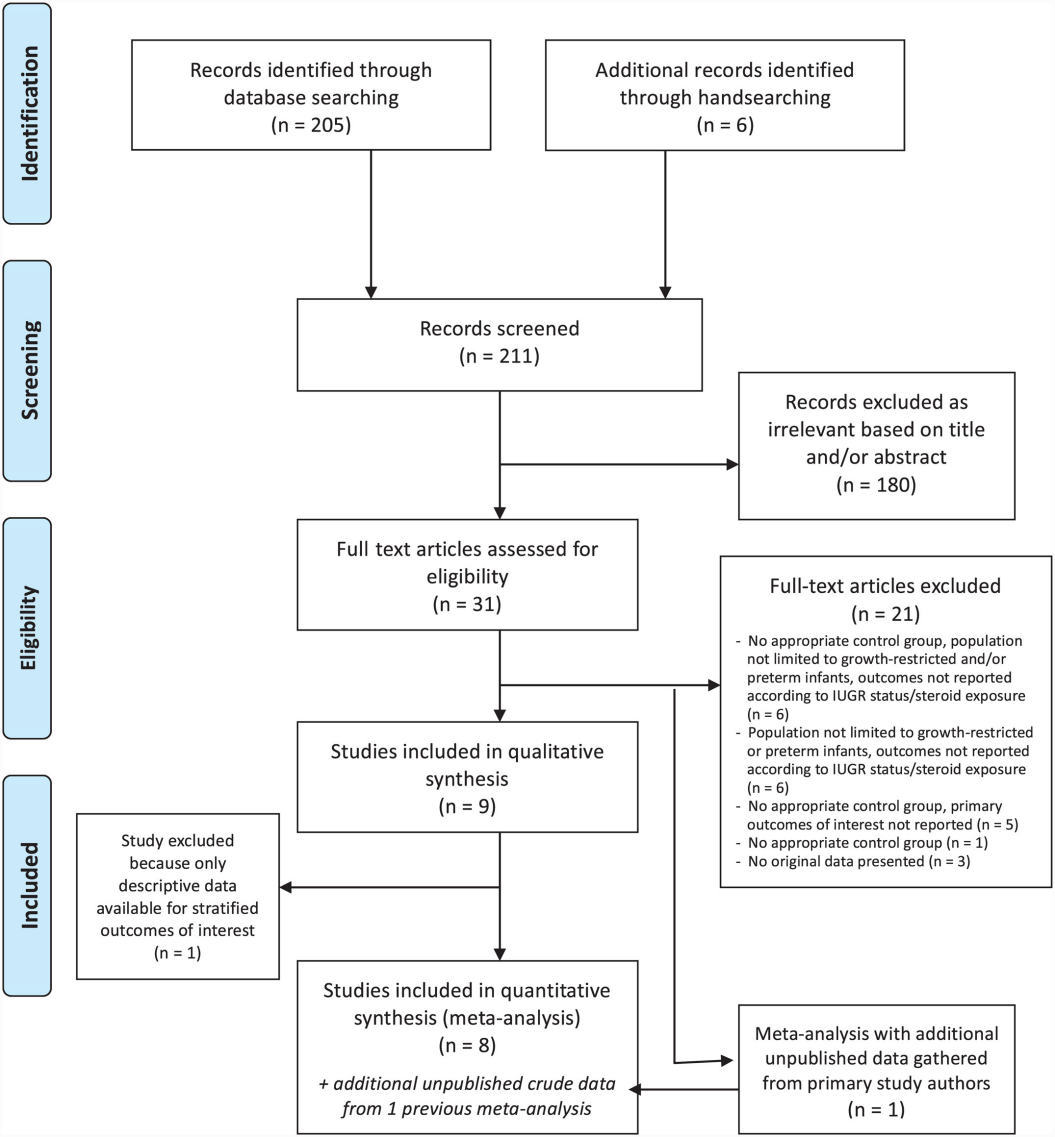
Flow diagram of search results and study selection for sub-question P4.

Our search identified 205 candidate citations from the electronic databases and 6 further articles by reviewing references lists of relevant papers and studies for sub-question P4. Full texts were obtained for 31 articles based on title and abstract review. After excluding ineligible studies, 9 studies met the inclusion criteria (4 including women with growth-restricted infants specifically and 5 including women with, more broadly, SGA infants^a^), out of which 8 studies were synthesized quantitatively (Fig 4), including data from a total of 2846 mother/newborn dyads [43,46–52]. One study was excluded from the meta-analysis because no exact data on number of SGA infants and no data on ORs for the SGA sub-population were reported [53]. The general characteristics of the eight studies included in the meta-analysis are summarized in Table 3. Additional unpublished data on one [51] of these eight primary studies was further extracted from a previous review paper identified through the search strategy [54]. Inclusion criteria for seven out of eight studies was gestational age less than 34 weeks, and one study was less than 35 weeks in Table 3. All studies were conducted in high-income countries with advanced maternal and newborn care including NICU.

**Table 3:** Basic characteristics of the included studies for sub-question P4 (women with SGA/growth-restricted infants). HC, histological chorioamnionitis; CC, clinical chorioamnionitis; Dexa, Dexamethasone; Beta, Betamethasone; RDS, respiratory distress syndrome; BPD, bronchopulmonary dysplasia; IHC, intrahepatic cholestasis; IVH, intraventricular haemorrhage; PVL, periventricular leukomalacia; NEC, necrotizing enterocolitis; PDA, patent ductus arteriosus; CLD, chronic lung disease.

Author, year	Study design	N (treatment, control)	Enrollment period	Location	Inclusion criteria	Exclusion criteria	Explicitly growth-restricted?	Antenatal corticosteroid course					Outcomes reported
								Drug	Dose (mg)	Interval (hrs)	ACS cut-off ^a^	Repeat?	
Di Lenardo et al., 1990	Retrospective cohort	72 (15, 57)	?	Italy	≤35 wks singletons	Twin pregnancies	No	Beta	12	24	?	?	RDS
Elimian et al., 1999	Retrospective cohort	220 (63, 157)	1990–1997	USA	≤1750g	None	No	Beta	12	24	≥2x	Yes	RDS; IVH/PVL; Major brain lesion; NEC; Neonatal sepsis; PDA; 5-min Apgar score <7; Postnatal surfactant exposure; Chorioamnionitis; Birth weight percentile; Neonatal death
Foix-L’Helias et al., 2005	Retrospective cohort	151 (55, 96)	1993–1996	France	24–31 wks	None	Yes	Beta/Dex	12/6	24/12	≥1x	Yes	RDS; BPD; Hospital death
Ley et al., 1997	Retrospective cohort	234 (117, 117)	1985–1984	Sweden	<33 wks	None	No	?	?	?	≥1x	?	RDS; IVH grade 3/PVH/PVL; Mortality
Schaap et al., 2001	Case-control	124 (62, 62)	1984–1999	Netherlands	26–31 wks live-born singletons	Maternal admittance <24 hrs before delivery, fetal death, fetal distress at admission, abruptio placentae, lethal congenital anomalies, congenital infections	Yes	Beta	12.5	24	≥2x	?	Birth weight>10^th^ percentile; RDS; ICH grades 3–4; Mechanical ventilation; BPD; Sepsis; Survival at discharge; Survival without disability or handicap at 2 years’ corrected age; Survival at long-term follow-up at school age
Spinillo et al., 1995	Prospective cohort	96 (32, 64)	1988–1993	Italy	24–34 wks liveborn	Triplets, pregnancies complicated by severe abruption or eclampsia	No	Beta/Dex	12/12	24/24	≥2x	?	RDS; IVH; Severe IVH; Neonatal death
Torrance et al., 2007	Retrospective cohort	142 (112, 30) /45 (34, 11)	1999–2003	Netherlands	<34 wks	Congenital, chromosomal, or syndromal abnormalities	Yes / No	Beta	12	24	≥2x	?	Gestational age at delivery; Birth weight; Apgar <5 at 1 min; Apgar <6 at 5 min; RDS; Surfactant treatment; Ventilation; CLD; Neonatal death
van Stralen et al., 2009	Retrospective cohort	88 (53, 34)	2001–2005	Netherlands	<34 wks, <1500g singletons	Multiple pregnancies, infants with major congenital abnormalities or infection and deliveries with insufficient data	Yes	Beta	12.5	24	≥2x		Median Apgar score at 5 min; Duration of mechanical ventilation; Hypotension; RDS; Mechanical ventilation; Administration of surfactant; Neonatal sepsis; PDA; BPD; NEC; Severe cerebral lesion; Neonatal death

^a^ Refers to the number of antenatal steroid doses used as a cut-off to determine antenatal corticosteroid administration as positive.

Table 4 summarizes the findings of the studies and the quality of the evidence for ACS use in women with growth-restricted infants, with pooled results shown separately for infants that could be classified as specifically growth-restricted (i.e., with evidence of placental insufficiency) and for the broader category of SGA (i.e., constitutionally small, with or without placental insufficiency) infants (see S4 File for Forest plots). The studies on populations designated as SGA do not report whether cases were examined for signs of placental insufficiency, thus likely resulting in heterogeneous SGA/growth-restricted populations. Applying a random-effects model did substantially divergent effect sizes or significance levels for any outcome measure.

**Table 4:** GRADE evidence profile for sub-question P4 (women with SGA/growth-restricted infants). SGA, small for gestational age; CI, confidence interval; OR, odds ratio; MD, mean difference. IVH, intraventricular haemorrhage; ICH, intracerebral haemorrhage; PVH, periventricular haemorrhage; PVL, periventricular leukomalacia.

Quality assessment							No. of patients		Effect		Quality
No. of studies	Study design	Risk of bias	Inconsistency	Indirectness	Imprecision	Other considerations	Antenatal corticosteroids	No antenatal corticosteroids	Relative (95% CI)	Absolute (95% CI)	
**Mode of delivery (caesarean section) (SGA)**											
1	observational studies	not serious	not serious	not serious	very serious ^a^	not serious	139/146 (95.2)%	19/19 (100.0)%	OR 0.48 (0.03 to 8.68)	0 fewer per 1000 (from 0 fewer to 0 fewer)	⊕○○○ VERY LOW
**Chorioamnionitis** (histological and/or clinical) **(SGA)**											
1	observational studies	serious ^b^	not serious	not serious	very serious ^a^	not serious	11/63 (17.5)%	34/157 (21.7)%	OR 0.77 (0.36 to 1.63)	41 fewer per 1000 (from 94 more to 126 fewer)	⊕○○○ VERY LOW
**Perinatal death** (fetal death or neonatal death) **(growth-restricted)**											
4	observational studies	not serious	not serious	not serious	serious ^c^	not serious	41/324 (12.7)%	33/179 (18.4)%	OR 0.81 (0.58 to 1.04)	30 fewer per 1000 (from 6 more to 68 fewer)	⊕○○○ VERY LOW
**Perinatal death** (fetal death or neonatal death) **(SGA)**											
6	observational studies	not serious	not serious	not serious	serious ^c^	serious ^e^	- ^d^	- ^d^	OR 0.78 (0.58 to 1.04)	1 fewer per 1000 (from 0 fewer to 0 fewer)	⊕○○○ VERY LOW
**Respiratory distress syndrome (growth-restricted)**											
4	observational studies	serious ^b^	not serious	not serious	serious ^c^	not serious	142/324 (43.8)%	88/179 (49.2)%	OR 0.81 (0.59 to 1.11)	52 fewer per 1000 (from 26 more to 128 fewer)	⊕○○○ VERY LOW
**Respiratory distress syndrome (SGA)**											
8	observational studies	serious ^b^	not serious	not serious	serious ^c^	not serious	- ^d^	- ^d^	OR 0.83 (0.66 to 1.05)	1 fewer per 1000 (from 0 fewer to 0 fewer)	⊕○○○ VERY LOW
**Surfactant use (growth-restricted)**											
1	observational studies	serious ^b^	not serious	not serious	very serious ^a^	not serious	19/53 (35.8)%	13/34 (38.2)%	OR 0.90 (0.37 to 2.20)	25 fewer per 1000 (from 121 more to 132 fewer)	⊕○○○ VERY LOW
**Surfactant use (SGA)**											
3	observational studies	serious ^b^	not serious	not serious	serious ^c^	not serious	81/262 (30.9)%	47/210 (22.4)%	OR 1.39 (0.85 to 2.28)	62 more per 1000 (from 27 fewer to 173 more)	⊕○○○ VERY LOW
**Major brain lesion (IVH, ICH, PVH, or PVL) (growth-restricted)**											
2	observational studies	not serious	not serious	not serious	very serious ^a^	not serious	12/116 (10.3)%	10/96 (10.4)%	OR 0.86 (0.35 to 2.10)	13 fewer per 1000 (from 65 fewer to 92 more)	⊕○○○ VERY LOW
**Major brain lesion (IVH, ICH, PVH, or PVL) (SGA)**											
5	observational studies	not serious	not serious	not serious	serious ^c^	serious ^e^	- ^d^	- ^d^	OR 0.57 (0.41 to 0.78)	1 fewer per 1000 (from 0 fewer to 0 fewer)	⊕○○○ VERY LOW
**Neonatal sepsis (growth-restricted)**											
2	observational studies	serious ^b^	not serious	not serious	very serious ^a^	not serious	45/115 (39.1)%	36/96 (37.5)%	OR 0.83 (0.44 to 1.58)	43 fewer per 1000 (from 112 more to 166 fewer)	⊕○○○ VERY LOW
**Neonatal sepsis (SGA)**											
3	observational studies	serious ^b^	not serious	not serious	serious ^c^	not serious	51/178 (28.7)%	45/253 (17.8)%	OR 1.00 (0.58 to 1.73)	0 fewer per 1000 (from 66 fewer to 94 more)	⊕○○○ VERY LOW
**Necrotizing enterocolitis (growth-restricted)**											
1	observational studies	serious ^b^	not serious	not serious	very serious ^a^	not serious	3/53 (5.7)%	2/34 (5.9)%	OR 0.96 (0.15 to 6.07)	2 fewer per 1000 (from 50 fewer to 216 more)	⊕○○○ VERY LOW
**Necrotizing enterocolitis (SGA)**											
3	observational studies	serious ^b^	not serious	not serious	very serious ^a^	not serious	4/116 (3.4)%	5/191 (2.6)%	OR 0.90 (0.22 to 3.76)	3 fewer per 1000 (from 20 fewer to 66 more)	⊕○○○ VERY LOW
**Chronic lung disease / Bronchopulmonary dysplasia (growth-restricted)**											
3	observational studies	serious ^b^	not serious	not serious	serious ^c^	not serious	47/211 (22.3)%	44/151 (29.1)%	OR 0.69 (0.43 to 1.13)	70 fewer per 1000 (from 14 more to 138 fewer)	⊕○○○ VERY LOW
**Chronic lung disease / Bronchopulmonary dysplasia (SGA)**											
4	observational studies	serious ^b^	not serious	not serious	serious ^c^	not serious	81/357 (22.7)%	50/170 (29.4)%	OR 0.69 (0.44 to 1.07)	71 fewer per 1000 (from 14 more to 139 fewer)	⊕○○○ VERY LOW
**Patent ductus arteriosus (growth-restricted)**											
1	observational studies	serious ^b^	not serious	not serious	very serious ^a^	not serious	10/53 (18.9)%	6/34 (17.6)%	OR 1.09 (0.35 to 3.32)	13 more per 1000 (from 27 fewer to 255 more)	⊕○○○ VERY LOW
**Patent ductus arteriosus (SGA)**											
2	observational studies	serious ^b^	not serious	not serious	serious ^c^	not serious	19/116 (16.4)%	16/191 (8.4)%	OR 1.70 (0.82 to 3.54)	51 more per 1000 (from 14 fewer to 161 more)	⊕○○○ VERY LOW
**Low birth weight** (<3^rd^ percentile for gestational age) **(SGA)**											
1	observational studies	not serious	not serious	not serious	very serious ^a^	not serious	63/146 (43.2)%	12/19 (63.2)%	OR 0.44 (0.16 to 1.19)	202 fewer per 1000 (from 39 more to 416 fewer)	⊕○○○ VERY LOW
**Duration of mechanical ventilation, days (growth-restricted)**											
2	observational studies	not serious	not serious	not serious	very serious ^a^	not serious	115	96	-	MD 1.09 higher (from 0.86 lower to 3.05 higher)	⊕○○○ VERY LOW
**Use of mechanical ventilation (growth-restricted)**											
2	observational studies	not serious	not serious	not serious	very serious ^a^	not serious	61/115 (53.0)%	45/96 (46.9)%	OR 1.24 (0.72 to 2.14)	54 more per 1000 (from 80 fewer to 185 more)	⊕○○○ VERY LOW
**Use of mechanical ventilation (SGA)**											
3	observational studies	not serious	not serious	not serious	serious ^c^	not serious	127/261 (48.7)%	56/115 (48.7)%	OR 1.04 (0.65 to 1.66)	10 more per 1000 (from 105 fewer to 125 more)	⊕○○○ VERY LOW
**Apgar score <7 at 5 min. (SGA)**											
2	observational studies	serious ^b^	not serious	not serious	serious ^c^	not serious	12/209 (5.7)%	26/176 (14.8)%	OR 0.76 (0.34 to 1.72)	31 fewer per 1000 (from 82 more to 92 fewer)	⊕○○○ VERY LOW
**Growth <10**^**th**^ **percentile in early childhood** (follow-up to school age) **(growth-restricted)**											
1	observational studies	not serious	not serious	not serious	serious ^f^	strong association	14/49 (28.6)%	3/42 (7.1)%	OR 5.20 (1.38 to 19.62)	214 more per 1000 (from 25 more to 530 more)	⊕⊕○○○ LOW

^a^ Estimate based on small sample size; wide confidence interval crossing the line of no effect.

^b^ Evidence based heavily or entirely on studies with design limitations including lack of adjustment for potential confounding factors.

^c^ Estimate based on wide confidence interval crossing the line of no effect.

^d^ Raw data unavailable for one of the included studies (only ORs and 95% CIs reported); generic inverse variance method used for meta-analysis.

^e^ Funnel plot suggests the presence of some degree of publication bias.

^f^ Estimate based on few events and/or small sample si

Two SGA studies reported on maternal outcomes; no significant difference was observed between groups in terms of histological (OR; 0.77, 95% CI 0.36–1.63; 1 study, 220 women) or clinical chorioamnionitis (OR: 0.83, 95% CI 0.16–4.20; 1 study, 220 women) or regarding CS delivery (OR 0.48, 95% CI 0.03–8.68; 1 study, 165 women). Perinatal mortality (fetal or neonatal death) did not differ significantly between groups in any of the intrauterine growth restriction studies (pooled OR: 0.81, 95% CI 0.58–1.14; 4 studies, 504 infants), nor in the majority of reports on SGA infants (pooled OR: 0.78, 95% CI 0.58–1.04; 6 studies, 958 infants), though the trend was toward a reduction in deaths in the groups receiving ACS. One SGA study reported a significant reduction in neonatal deaths in steroid-treated SGA infants, but provided no specific data on this metric [53].

No significant difference in RDS of any severity was seen across the intrauterine growth restriction studies (pooled OR: 0.81, 95% CI 0.59–1.11; 4 studies, 504 infants), nor in the majority of reports on SGA infants, though pooled analyses revealed a clear trend favouring ACS (pooled OR: 0.83, 95% CI 0.66–1.05, 8 studies, 1126 infants). The one study excluded from meta-analysis found a significant reduction in RDS risk in ACS-treated SGA infants; however, this ACS-associated RDS risk reduction was smaller in SGA infants as compared to that observed in appropriately-grown infants [53]. Incidence of major brain lesion (IVH grade 3–4, ICH, PVH, PVL, ventricular dilation, or CPL) did not differ between treated and untreated growth-restricted infants (pooled OR: 0.86, 95% CI 0.35–2.10; 2 studies, 211 infants). For SGA infants, however, such outcomes were significantly improved in those receiving ACS (pooled OR: 0.57, 95% CI 0.41–0.78; 5 studies, 761 infants). Neither use of surfactants (pooled OR: 1.39, 95% CI 0.85–2.28; 3 studies, 472 infants), neonatal sepsis (pooled OR: 1.00, 95% CI 0.58–1.73; 3 studies, 431 infants), nor NEC (pooled OR: 0.90, 95% CI 0.22–3.76; 2 studies, 307 infants) differed between ACS treated and untreated groups across both growth-restricted and SGA infants.

No significant difference in the occurrence of PDA (pooled OR: 1.70, 95% CI 0.82–3.54; 2 studies, 307 infants) or CLD/BPD (pooled OR: 0.69, 95% CI 0.44–1.07; 4 studies, 527 infants) was found between treated and untreated neonates across growth-restricted and SGA infants. Likewise, no significant difference was observed for low (< 3^rd^ centile for gestational age) birth weight (OR: 0.44, 95% CI 0.16–1.19; 1 study, 165 infants), duration of mechanical ventilation (pooled MD: 1.09, 95% CI -0.86–3.05; 2 studies, 211 infants), Apgar <7 at 5 minutes (pooled OR: 0.76, 95% CI 0.34–1.72; 2 studies, 385 infants), hypotension (OR: 2.29, 95% CI 0.75–7.03; 1 study, 87 infants), or use of mechanical ventilation (pooled OR: 1.04, 95% CI 0.65–1.65; 3 studies, 376 infants) between treated and untreated groups across both growth-restricted and SGA subgroups.

As far as long-term outcomes following ACS treatment, one study [49], undertaken in a growth-restricted population, found significantly higher rates of survival without handicap at two years’ corrected age in infants treated with ACS (82% vs. 65% in the treated and untreated groups, respectively [OR: 2.55, 95% CI: 1.11–5.87]; 115 infants). When followed up at school age, however, no significant difference in survival was observed (OR: 2.50, 95% CI: 0.70–10.90; 91 infants). Additionally, study authors found no difference in incidence of abnormal behaviour between groups (OR: 0.90, 95% CI: 0.40–2.10; 91 infants). Those exposed to ACS treatment were, however, significantly more likely to exhibit physical growth below the 10^th^ percentile at school age (OR: 5.10, 95% CI: 1.40–23.8) [49].

Quality of the evidence was graded as very low across all measured outcomes *except* childhood growth beneath the 10^th^ percentile, for which the evidence quality was graded as low. See S5 File for a full summary of the risk of bias assessment undertaken for each of the included studies.

## Discussion

This systematic review highlights the paucity of rigorous evidence on use of ACS therapy in four special populations of pregnant women at risk of imminent preterm birth: women with diabetes mellitus, those undergoing elective CS in late preterm, those with chorioamnionitis, and those with SGA/growth-restricted infants. Across these understudied but important subgroups, no RCTs and, in the case of two subgroups (diabetic mothers and those undergoing elective CS), neither RCTs nor observational studies were available. Despite low to very low confidence in the estimates of effect obtained from available observational studies on use of ACS for mitigating adverse outcomes associated with preterm delivery in women with chorioamnionitis and SGA/growth-restricted fetuses, this review compiles best available evidence for these understudied subgroups. Supplemented with indirect evidence available from existing RCTs relevant to our questions of interest, review findings can be used to draw tentative conclusions on risk-benefit balances from which to operate while awaiting a more robust evidence base.

### ACS for improving preterm birth outcomes with chorioamnionitis

This review updates and provides a more solid foundation for the findings of the former review conducted by Been et al [24], incorporating a new key study not previously reviewed [40]. Based on the nine included studies and the quantitative synthesis of eight of these studies, ACS use appears to be safe and to reduce adverse neonatal outcomes following preterm birth associated with chorioamnionitis, whether histologically or clinically diagnosed—though, in the case of clinically apparent chorioamnionitis, the evidence is less robust and more susceptible to bias. In patients with histologically diagnosed chorioamnionitis, ACS administration was linked to significant reductions in mortality, RDS, PDA, all degrees of IVH, and severe IVH. In patients with clinically diagnosed chorioamnionitis, meanwhile, ACS therapy was correlated with reductions only in IVH and PVL.

From a practical perspective, assessments of ACS efficacy based on histological cases of chorioamnionitis may seem of limited clinical relevance. At the critical juncture for ACS treatment decision making, after all, clinicians will not have access to information on placental histology. Nevertheless, the beneficial effects observed with ACS administration in pregnancies characterized by histological chorioamnionitis imply that treatment may in fact be safe and effective, regardless of the presence or absence of subclinical inflammation.

From a theoretical perspective, valid concerns persist. Given the immunosuppressive properties of corticosteroids, the risk of activating latent infections through their use in pregnant women cannot be dismissed outright, and quality evidence to conclusively refute or confirm such risk is still lacking. However, observational evidence compiled for this review suggests that use of ACS confers certain benefits to the preterm infant without increasing such potential harms as neonatal sepsis. Maternal outcomes, meanwhile, were not reported by any of the reviewed studies, but indirect evidence from randomized trials of ACS therapy in general populations of women at risk of preterm birth suggests that exacerbation of existing infectious morbidity in the mother is unlikely; comparable risks of both chorioamnionitis and puerperal sepsis were observed in corticosteroid-treated and untreated women [4].

At the same time, interpretation of the evidence base for this question must be tempered with recognition of its limitations. Perhaps most importantly, included studies do not indicate *when* the ACS was administered relative to the diagnosis of maternal intrauterine infection. Importantly, corticosteroids may well have been administered *before* the emergence of clinically apparent chorioamnionitis in most cases [22], rendering outcome measures less useful in guiding their application. On the other hand, ACS may have been administered just before delivery, such as, for example, in cases where a clinical diagnosis of chorioamnionitis was made. Where the elapsed time was insufficient for the ACS to take full effect, this could explain the lack of benefit observed in certain neonatal outcomes such as neonatal mortality and RDS in the context of clinical chorioamnionitis. Thus, although the current meta-analysis showed improved short-term neurological outcomes in infants with clinical chorioamnionitis, prudence is recommended in extrapolating these findings directly to the clinical situation. Overall, there is a need for studies on maternal and long-term child outcomes, as well as randomized clinical trials to guide practice in this subgroup.

### ACS for improving preterm birth outcomes with SGA/growth-restricted infants

From the available studies, administration of ACS to growth-restricted preterm infants did not improve neonatal mortality or morbidity, though some positive effects in terms of childhood health status were observed. In particular, meta-analysis revealed no reduction in RDS incidence or incidence of major brain lesion after ACS therapy in growth-restricted infants. As far as follow-up into childhood, a reduction in handicaps has been reported in steroid-treated growth-restricted infants at two years of age [49]. However, data on such risk into school age is lacking from this cohort, while no differences were observed in behaviour among ACS-treated infants followed into school age and physical growth below the 10^th^ percentile was significantly more frequent in the treatment group [49].

Given the chronic intrauterine stress to which the growth-restricted infant has already been subjected and the prolonged stimulation of the adrenal gland thus stimulated, intrauterine growth restriction itself may effectively lead to enhanced fetal lung maturation as well as accelerated development of the central nervous system [55]. Through such stabilizing mechanisms, growth-restricted infants as a group may have a lower baseline susceptibility to morbidities like RDS and brain lesions, as a result of which exogenous corticosteroid administration in this particular group may have no additional benefit, at least in the short term [25,27].

In this review, we found an improvement in major brain lesions among SGA infants—a trend not identified in the previous review by Torrance et al [54]. However, overall, the benefit of ACS therapy in SGA infants remains unclear from our review of the current literature, possibly due to heterogeneity in the populations and treatment regimens studied. Namely, as the included studies on SGA infants did not report whether cases were examined for signs of placental insufficiency (as indicated by abnormal umbilical blood flow Doppler studies and/or placental pathology), a heterogeneous SGA/growth-restricted population is likely in this evidence. Moreover, two of the designated SGA studies did not strictly define the type of steroid treatment used or designate completion of ACS therapy as a requirement for inclusion in the treatment group [48,53,54].

Overall, there is thus insufficient evidence to conclude on the benefits or harms of ACS therapy in women whose infants were growth-restricted in-utero or who are likely to deliver SGA preterm infants. Routine use of ACS in growth-restricted infants should thus be re-evaluated, as the potential detrimental side effects of steroids on growth are specifically unwarranted in this already growth-restricted group. An RCT is merited to clarify whether treatment brings any added benefit in growth-restricted infants and to address further questions regarding ACS treatment of SGA infants.

### ACS for improving preterm birth outcomes with maternal diabetes mellitus

Our review identified no eligible studies on preterm birth outcomes following ACS therapy in pregnancies complicated by maternal diabetes mellitus. However, while there is no direct evidence to clearly demonstrate the benefits or harms of ACS in this population of women, available findings suggest a hyperglycaemic effect of corticosteroids in pregnant women [56] [57].

Direct evidence for ACS administration in diabetic pregnancies is lacking; however, available indirect evidence suggests the prudence of a cautious approach to use of ACS in such cases, with close monitoring and treatment for glycaemic control essential at all stages [58]. Without appropriate monitoring and adjustment of insulin dosage, this, in turn, may create an increased risk of severe dysregulation with ketoacidosis. With maternal hyperglycaemia, fetal lung development may also be compromised [59], potentially offsetting any benefits of ACS therapy to the infant. However, direct evidence for this is lacking. Pending better data, the presence of maternal diabetes in pregnancy is not a reason to withhold antenatal corticosteroids where there is an imminent risk of preterm birth, given the significant health benefits to the infant.

### ACS for improving preterm birth outcomes with elective CS in late preterm

Direct evidence regarding effectiveness and safety of ACS therapy for reducing adverse maternal and newborn outcomes in women undergoing elective CS in late preterm is lacking. However, indirect evidence from a single RCT [60,61] suggests that prophylactic betamethasone preceding elective CS *at term* (i.e., ≥ 37 weeks gestation) may facilitate significant reductions in admissions to neonatal special care units for respiratory complications, though such reductions were not in evidence for clinical measures of respiratory morbidity. No adverse effects were observed in health or behaviour of children born following a single course of antenatal betamethasone administered at term, nor did such treatment reduce the prevalence of asthma or allergy following elective CS. Some evidence of detrimental impact on academic ability at school age was, however, found for children of treated mothers [61].

These findings may be regarded as indirect evidence for the question of interest, though caution should be exercised in interpreting the potential benefits and harms, given the subjective quality of the measured outcomes and the lack of blinding in the trial. Additionally, it should be noted that respiratory morbidity in the context of term elective caesarean births may exhibit a different pathophysiology compared with preterm birth, likely due to the absence of the physiological catecholamine surge [62,63] and the presence of fluid retention in the lungs [64]. Hence, particular caution should be exercised in extrapolating findings from term births to cases of preterm delivery. An overall lack of evidence thus precludes any conclusion being drawn on the benefits or harms of using ACS in women undergoing elective CS in later preterm.

### Strengths and limitations

Primary strengths of the present review include the comprehensive search strategy used, inclusion of studies reported in languages other than English, use of GRADE methodology to assess the quality of included studies, and in-depth assessment of factors influencing confidence in the results across studies and questions. By implementing such an inclusive search strategy across the major databases of medical literature, we minimized bias potentially introduced in the search process and minimized the likelihood of inadvertently omitting important published data, while the hand searching subsequently undertaken sought to identify any relevant unpublished findings, as far as possible. Further support for the validity of the results can be found in the absence of statistical indications for publication bias or study heterogeneity. Finally, the meta-analyses conducted contribute important data on subgroups and outcomes of ACS therapy that are not currently available from RCTs.

At the same time, limitations stemming from the nature of the evidence itself cannot be ignored in interpreting the results and should be used to guide future research. Overall, the review was constrained by the dearth of eligible studies. Moreover, several sources of potential bias reduce confidence in the estimates of effect. The considerable size of individual cohorts notwithstanding, intra-study differences in the inclusion and exclusion criteria may introduce bias. In particular, studies varied importantly in the specific type of corticosteroid administered, in drug dosages and timing, and in whether or not multiple or incomplete courses of ACS were permitted in which group, treatment or control. Similarly, differences in diagnostic criteria related to both the defining subgroup condition and outcome definitions may have been a source of further variation across studies. Unfortunately, incorporating such a variety of study types may inherently lead to bias, though comparison of random-effects and fixed-effects models suggest that the effect of heterogeneity was limited in the present review.

Perhaps most importantly, this review is limited by its reliance on studies, where available for a maternal subgroup, from high-income and Western countries, especially given that over 60% of all preterm births are occurring in Africa and South Asia [65]. Indeed, as noted in a series of editorials published in the *Lancet* and *Lancet Global Health* [66–68], the majority of evidence for general populations, too, is characterized by this same limitation. Results from Althabe *et al*.’s [69] international, cluster-randomized trial of an intervention including measures to implement appropriate use of ACS for reducing neonatal mortality from preterm births in low- and middle-income settings has underlined that context matters, prompting renewed concerns about the potential consequences of global scale-up. Additionally, reporting on maternal outcomes is notably sparse and on long-term follow-up into adulthood entirely lacking for children in any of the reviewed subgroups, which is important because antenatal treatment is hypothesized to be a key mechanism underlying the fetal origins of adult disease hypothesis [33]. Further studies undertaken in low-income and hard-to-reach populations are needed–especially in places where diagnostic capacity, standards of care, and capacity to handle complications are likely to be lower. Furthermore, subgroup-specific analyses of maternal and long-term child outcomes are needed before a clear picture of the risk-benefit ratio of ACS for preterm delivery can be achieved.

## Conclusions

Current evidence is not sufficient to guide administration of ACS to pregnant women with pregestational or gestational diabetes mellitus, clinical or subclinical chorioamnionitis at risk of imminent preterm birth, and in those at risk of preterm birth of a growth-restricted infant. The findings of this review further highlight the dearth of research on the effectiveness and safety of ACS for diabetic mothers at risk of imminent preterm birth and for women undergoing elective caesarean birth in late preterm, for which direct evidence is lacking. High-quality, population-based studies across a range of resource and population contexts and with longitudinal follow-up will be required to establish the short-term effectiveness and long-term safety of ACS for improving both maternal and child outcomes across these four special populations of women in diverse settings.

This systematic review conforms to the Preferred Reporting Items for Systematic Reviews and Meta-Analyses (PRISMA) statement. A PRISMA checklist is accordingly provided as S6 File.

## Endnotes

^**a**^ Given the paucity of studies on the growth-restricted infant specifically, we also decided to include reports on SGA infants (i.e., those having a fetal weight below the 10^th^ percentile for gestational age based on abdominal and head circumference, or a birth weight below the 10^th^ percentile for gestational age). Growth-restricted infants, meanwhile, are a subgroup of SGA infants whose growth has been restricted due to placental insufficiency, as diagnosed by abnormal Doppler examination of the umbilical artery or pathological examination of the placenta. Overall, it appears that 76% of SGA infants are truly growth-restricted, the remaining 24% being constitutionally/genetically small [70,71].

## Supporting Information

S1 File Database-specific search terms and strategies. (DOCX)

S2 File Forest plots for sub-question P3 (women with chorioamnionitis) meta-analyses. (DOCX)

S3 File Risk of bias assessments for sub-question P3 (women with chorioamnionitis). (DOCX)

S4 File Forest plots for sub-question P4 (women with SGA/growth-restricted infants) meta-analyses. (DOCX)

S5 File Risk of bias assessments for sub-question P4 (women with SGA/growth-restricted infants). (DOCX)

S6 File PRISMA checklist. (DOC)

## Abbreviations

ACS: Antenatal corticosteroids
RDS: Respiratory distress syndrome
RR: Risk ratio
95% CI: 95% Confidence interval
CVH: Cerebroventricular haemorrhage
NEC: Necrotizing enterocolitis
CS: Caesarean section
RCT: Randomized controlled trial
MOOSE: Meta-analysis Of Observational Studies in Epidemiology
WHO: World Health Organization
CINAHL: Cumulative Index to Nursing and Allied Health Literature
CENTRAL: Cochrane Register of Controlled Trials
ISRCTN: International Standard Randomised Controlled Trial Number Register
ICTRP: International Clinical Trials Registry Platform
RevMan 5: Review Manager version 5
NOS: Newcastle-Ottawa Scale
RoBANS: Risk of Bias Assessment tool for Non-randomized Studies
OR: Odds ratio
MD: Mean difference
GDT: Guideline Development Tool
IVH: Intraventricular haemorrhage
PVL: Periventricular leukomalacia
CLD: Chronic lung disease
BPD: Bronchopulmonary dysplasia
PDA: Patent ductus arteriosus
GRADE: Grading of Recommendations Assessment, Development and Evaluation
