# Supplementary material for: Antenatal Corticosteroids for Reducing Adverse Maternal and Child Outcomes in Special Populations of Women at Risk of Imminent Preterm Birth: A Systematic Review and Meta-Analysis
Source: PLoS One. 2016 Feb 3;11(2):e0147604. doi: 10.1371/journal.pone.0147604 (PMC4740425; doi:10.1371/journal.pone.0147604)
Supplement: S1 File — (DOCX) [file pone.0147604.s001.docx]

**Additional file 1 - Database-specific search terms and strategies**

***Note: P1*** *designates hits for antenatal corticosteroid therapy used in pregestational and gestational diabetic women at risk of imminent preterm birth.* ***P2*** *designates hits for antenatal corticosteroid therapy used in women undergoing elective caesarean birth in late preterm (35 weeks to <36 weeks + 6 days).* ***P3*** *designates hits for antenatal corticosteroid therapy used in women with intrapartum bacterial infections (e.g., chorioamnionitis, systemic infections) who are at risk of imminent preterm birth.* ***P4*** *designates hits for women with growth-restricted fetuses at risk of preterm birth.*

**Search strategy for MEDLINE (conducted February 13, 2014)**

**ID Query/Search terms Hits**

1 exp *Adrenal Cortex Hormones/ad, tu 51237

2 exp *Adrenal Cortex Hormones/ and (ci or de of dt).fs. 12129

3 exp Adrenal Cortex Hormones/ae, po, to 26632

4 or/1-3 74819

5 exp Pregnancy/ 701821

6 exp Pregnancy Outcome/ 39103

7 Fetal Death/ 22788

8 Maternal Death/ 54

9 Obstetric Labor Complications/ 14748

10 exp Obstetric Labor, Premature/ 17483

11 Pregnancy, Prolonged/ 2232

12 Fetus/ 67375

13 exp Infant, Newborn/ 488054

14 Prenatal Care/ 19889

15 exp Fetal Development/ 72793

16 exp Birth Weight/ 32985

17 Prenatal Exposure Delayed Effects/ 19379

18 or/5-17 1089224

19 4 and 18 4579

20 limit 19 to (biography or case reports or comment or congresses or

consensus development conference or consensus development

conference, nih or editorial or guideline or historical article or interactive

tutorial or interview or introductory journal article or lectures or news or

newspaper article or overall or patient education handout or practice

guideline or "review" or "scientific integrity review" or systematic

reviews) 1432

21 limit 20 to meta analysis 69

22 20 not 21 1363

23 19 not 22 3216

24 limit 23 to humans 2345

25 ("*corticosteroid" or "*corticoid").mp. 80067

26 (pregnan* or labor or labour or gestation* or delivery* or preterm* or

fetus or fetal or baby or babies or newborn* or neonat* or antenat* or

prenat* or birth*).mp. 1768197

27 25 and 26 7035

28 MEDLINE.st. 21110122

29 27 not 28 300

30 (biograph* or case report* or comment or congress* or conference* or

editor* or tutorial* or interview* or lecture* or news* or handout* or

guideline* or (review* not (meta analys* or metaanalys*))).mp. 5559139

31 29 not 30 185

32 exp Diabetes Mellitus/ 306590

33 exp Hyperglycemia/ 25349

34 or/32-33 318409

35 34 and 18 18236

36 exp Diabetes, Gestational/ 6669

37 Pregnancy in Diabetics/ 9698

38 or/36-37 15389

39 or/5-17 1089224

40 38 and 39 15340

41 or/35,40 23015

42 4 and 41 99

43 limit 42 to (biography or case reports or comment or congresses or

consensus development conference or consensus development

conference, nih or editorial or guideline or historical article or interactive

tutorial or interview or introductory journal article or lectures or news or

newspaper article or overall or patient education handout or practice

guideline or "review" or "scientific integrity review" or systematic

reviews) 46

44 limit 43 to meta analysis 0

45 43 not 44 46

46 42 not 45 53

47 limit 46 to humans 43

48 diabet*.mp. 455793

49 31 and 48 11

50 or/47,49 54

51 remove duplicates from 50 54

52 exp epidemiologic study characteristics as topic/ 1953367

53 (trial* or comparative or meta analysis or metaanalysis or multicenter or

observational or randomized or randomised or rct or cct or cohort or

cross sectional or longitudinal or evaluation or prospective or

retrospective or control*).mp. 6400082

54 or/52-53 6655110

55 51 and 54 **29**

56 51 not 55 **25 P1 (29+25)**

57 exp Cesarean Section/ 34591

58 (cesarean or cesarian or caesarean or caesarian).mp. 53534

59 or/57-58 53534

60 or/24,31 2530

61 60 and 59 87

62 remove duplicates from 61 87

63 62 and 54 **56**

64 62 not 63 **31 P2 (56+31)**

65 exp "Bacterial Infections and Mycoses"/ 1144595

66 Pregnancy Complications, Infectious/ 30512

67 or/65-66 1144595

68 24 and 67 157

69 (infect* or chorioamnionitis).mp. 1609705

70 31 and 69 19

71 or/68,70 176

72 remove duplicates from 71 175

73 72 and 54 **91**

74 72 not 73 **84 P3 (91+84)**

75 exp *Fetal Development/ 9314

76 (growth adj3 restrict*).mp. 9423

77 or/75-76 18318

78 24 and 77 105

79 ((fetal or fetus or baby or babies or restricted) adj3 (development or

growth or maturity or weight)).mp. 62442

80 31 and 79 10

81 or/78,80 115

82 remove duplicates from 81 113

83 82 and 54 **87**

84 82 not 83 **26 P4 (87+26)**

**Search strategy for EMBASE (conducted February 13, 2014)**

**ID Query/Search terms Hits**

#1 'corticosteroid'/exp/mj/dd_do,dd_cm,dd_dt,dd_ad,dd_to,dd_ct,dd_it 107592

#2 'corticosteroid'/exp/dd_ae 51958

#3 #1 OR #2 140064

#4 #3 AND 'human'/de 122240

#5 #4 AND [embase]/lim NOT [medline]/lim 27496

#6 'parameters concerning the fetus, newborn and pregnancy'/exp 233535

#7 'fetus death'/exp 29155

#8 'labor complication'/exp 131473

#9 'prolonged pregnancy'/de 2534

#10 'fetus'/de 161534

#11 'newborn'/de 464423

#12 'prenatal care'/exp 107055

#13 'prenatal development'/exp 180462

#14 'prenatal exposure'/de 15438

#15 #6 OR #7 OR #8 OR #9 OR #10 OR #11 OR #12 OR #13 OR #14 991052

#16 #5 AND #15 687

#17 'editorial'/de OR 'erratum'/exp OR 'note'/de OR 'review'/de 3061383

#18 'meta analysis'/exp 75918

#19 #17 NOT #18 3030271

#20 #16 NOT #19 415

#21 'case report'/exp 1896062

#22 #20 NOT #21 334

#23 'diabetes mellitus'/exp 583007

#24 'hyperglycemia'/de 58448

#25 #23 OR #24 608033

#26 #22 AND #25 **39 P1**

#27 'cesarean section'/de 60032

#28 #22 AND #27 **13 P2**

#29 'infection'/exp 2533874

#30 'chorioamnionitis'/de 4332

#31 #29 OR #30 2536210

#32 #22 AND #31 **82 P3**

#33 'prenatal development'/exp/mj 55811

#34 #22 AND #33 **20 P4**

**Search strategy for CINAHL (conducted February 14, 2014)**

**ID Query Limiters/Expanders Hits**

S1 (MM "Adrenal Cortex Hormones+/AD/DE/TU") 4,520

S2 (MH "Adrenal Cortex Hormones+/AE") 2,657

S3 S1 or S2 6,438

S4 (MH "Pregnancy+") 118,430

S5 (MH "Expectant Mothers") 2,240

S6 (MH "Pregnancy Outcomes") 11,951

S7 (MH "Perinatal Death") 4,285

S8 (MH "Maternal Mortality") 3,044

S9 (MH "Labor Complications+") 5,833

S10 (MH "Labor, Premature") 2,177

S11 (MH "Pregnancy, Prolonged") 218

S12 (MH "Fetus+") 17,511

S13 (MH "Infant, Newborn+") 81,600

S14 (MH "Prenatal Care") 9,435

S15 (MH "Fetal Development+") 12,517

S16 (MH "Birth Weight") 5,504

S17 (MH "Prenatal Exposure Delayed Effects") 2,494

S18 S4 or S5 or S6 or S7 or S8 or S9 or S10 or S11 or

S12 or S13 or S14 or S15 or S16 or S17 186,570

S19 S3 and S18 545

S20 S19 Limiters - Human 261

S21 S20 Limiters - Research Article; Exclude MEDLINE records 21

S22 (MH "Metabolic Diseases") OR (MH "Diabetes Mellitus+") 93,327

S23 (MH "Hyperglycemia") 5,117

S24 (MH "Pregnancy in Diabetes+") 4,227

S25 S22 or S23 or S24 95,535

S26 S21 and S25 **0** **P1**

S27 (MH "Cesarean Section+") 9,892

S28 S21 and S27 **0** **P2**

S29 (MH "Bacterial and Fungal Diseases (Non-Cinahl)+") 151,518

S30 S21 and S29 **3** **P3**

S31 (MM "Fetal Development+") 3,102

S32 restrict* N3 (growth or development or matur*) 1,497

S33 S31 or S32 4,507

S34 S21 and S33 **1** **P4**

**Search strategy for Cochrane Library (conducted February 14, 2014)**

**ID Query/Search terms Hits**

#1 MeSH descriptor: [Adrenal Cortex Hormones] explode all trees 11158

#2 *corticosteroid* or *corticoid* 14856

#3 #1 or #2 20492

#4 MeSH descriptor: [Pregnancy] explode all trees 5585

#5 pregnan* or labor or labour 30661

#6 MeSH descriptor: [Pregnancy Outcome] explode all trees 2633

#7 stillbirth or livebirth 548

#8 MeSH descriptor: [Fetal Death] explode all trees 205

#9 MeSH descriptor: [Maternal Death] explode all trees 3

#10 MeSH descriptor: [Obstetric Labor, Premature] explode all trees 1056

#11 MeSH descriptor: [Pregnancy, Prolonged] explode all trees 113

#12 MeSH descriptor: [Obstetric Labor Complications] this term only 409

#13 MeSH descriptor: [Fetus] this term only 319

#14 fetus or fetal 7551

#15 MeSH descriptor: [Infant, Newborn] explode all trees 12633

#16 infant* or newborn* or neonate* or baby or babies 40300

#17 MeSH descriptor: [Prenatal Care] explode all trees 1001

#18 prenatal or antenatal or perinatal 7591

#19 MeSH descriptor: [Fetal Development] explode all trees 1887

#20 matur* or immatur* or prematur* 16051

#21 MeSH descriptor: [Birth Weight] explode all trees 1072

#22 MeSH descriptor: [Prenatal Exposure Delayed Effects] explode all trees 233

#23 gestation* or birth* or offspring 19158

#24 #4 or #5 or #6 or #7 or #8 or #9 or #10 or #11 or #12 or #13 or #14 or

#15 or #16 or #17 or #18 or #19 or #20 or #21 or #22 or #23 74076

#25 #3 and #24 2710

#26 MeSH descriptor: [Diabetes Mellitus] explode all trees 15412

#27 diabet* or dm 44481

#28 MeSH descriptor: [Hyperglycemia] explode all trees 1276

#29 hyperglycem* 2503

#30 MeSH descriptor: [Diabetes, Gestational] explode all trees 318

#31 MeSH descriptor: [Pregnancy in Diabetics] explode all trees 198

#32 #26 or #27 or #28 or #29 or #30 or #31 45469

#33 #25 and #32 607

#34 handsrch 164394

#35 #33 and #34 **7** **P1**

#36 MeSH descriptor: [Cesarean Section] explode all trees 2315

#37 cesarean or cesarian or caesarean or caesarian 5886

#38 #36 or #37 5886

#39 #25 and #38 134

#40 #39 and #34 **1** **P2**

#41 MeSH descriptor: [Bacterial Infections and Mycoses] explode all trees 27364

#42 infect* 67359

#43 MeSH descriptor: [Pregnancy Complications, Infectious]

explode all trees 1039

#44 chorioamnionitis 408

#45 #41 or #42 or #43 or #44 72860

#46 #25 and #45 1091

#47 #46 and #34 **22 P3**

#48 growth near restrict* 460

#49 #25 and #48 60

#50 #49 and #34 **59 P4**

**Search strategy for POPLINE (conducted February 14, 2014)**

**Query/Search terms Hits**

*cortico* AND (labor OR labour OR prematur* OR immatur* OR

matur*) AND (diaebet* OR DM OR hyperglycem*) **1** **P1**

*cortico* AND (labor OR labour OR prematur* OR immatur* OR

matur*) AND (elective caesarean) **0** **P2**

*cortico* AND (labor OR labour OR prematur* OR immatur* OR

matur*) AND (infect*) **10** **P3**

*cortico* AND restrict* AND growth **3** **P4**

**Search strategy for WHO Global Health Library, Regional Index (conducted February 14, 2014)**

**Query/Search terms Hits**

*cortico* AND (labor OR labour OR prematur* OR immatur* OR

matur*) AND (diaebet* OR DM OR hyperglycem*) **5** **P1**

*cortico* AND (labor OR labour OR prematur* OR immatur* OR

matur*) AND (elective caesarean) **1** **P2**

*cortico* AND (labor OR labour OR prematur* OR immatur* OR

matur*) AND (infect*) **38** **P3**

*cortico* AND restrict* AND growth **9** **P4**
