# Supplementary material for: Antenatal Corticosteroids for Reducing Adverse Maternal and Child Outcomes in Special Populations of Women at Risk of Imminent Preterm Birth: A Systematic Review and Meta-Analysis
Source: PLoS One. 2016 Feb 3;11(2):e0147604. doi: 10.1371/journal.pone.0147604 (PMC4740425; doi:10.1371/journal.pone.0147604)

**Additional file 2 – Forest plots for sub-question P3 (women with chorioamnionitis) meta-analyses**

**1.1 Neonatal death**


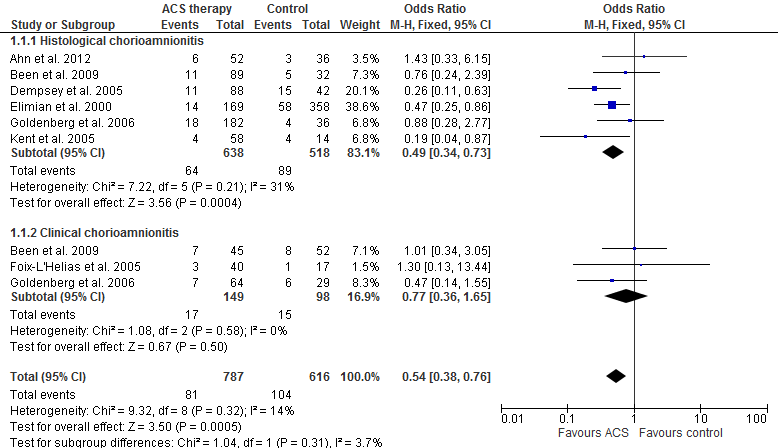


**1.2 Respiratory distress syndrome (RDS)**


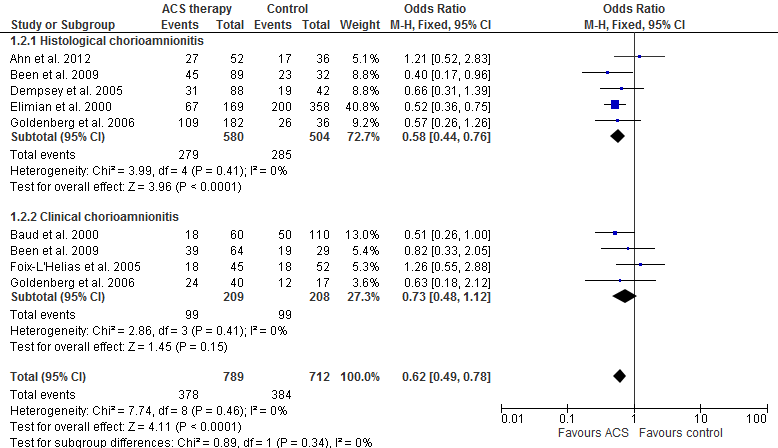


**1.3 Surfactant use**


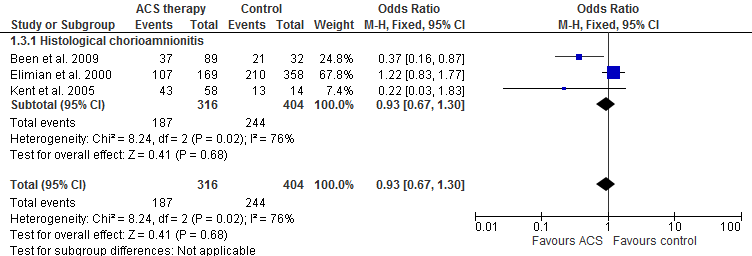


**1.4 Intraventricular haemorrhage (IVH)**


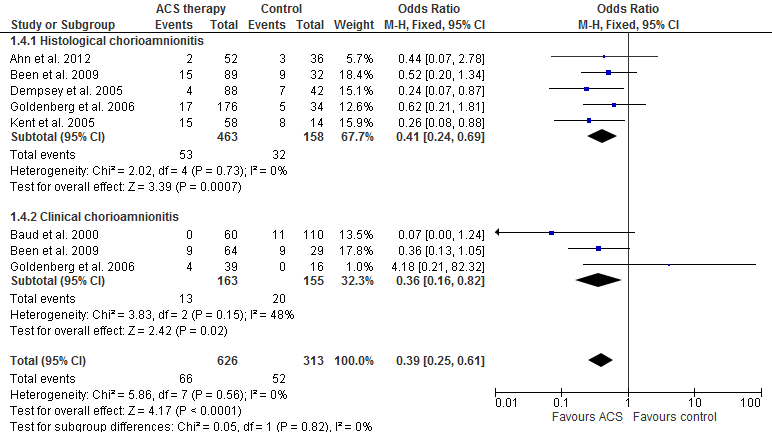


**1.5 Severe IVH (grade 3-4)**


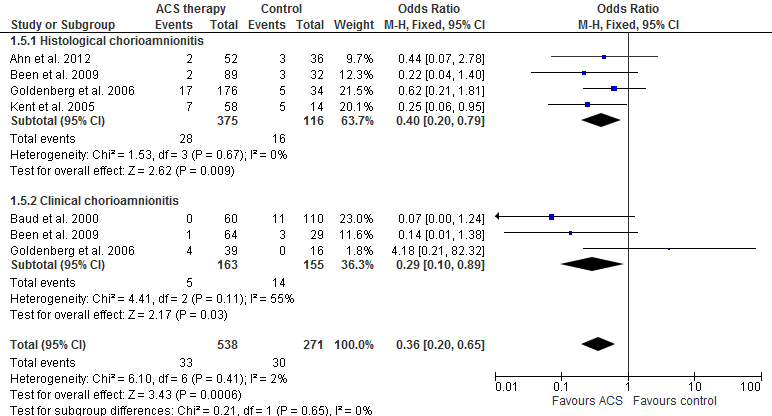


**1.6 Periventricular leukomalacia (PVL)**


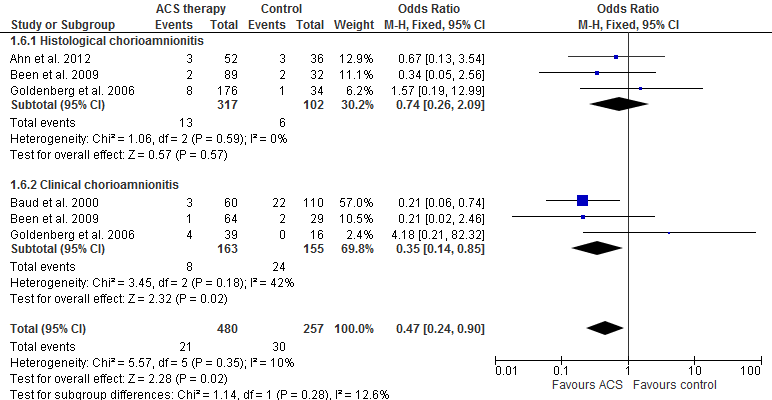


**1.7 Neonatal sepsis**


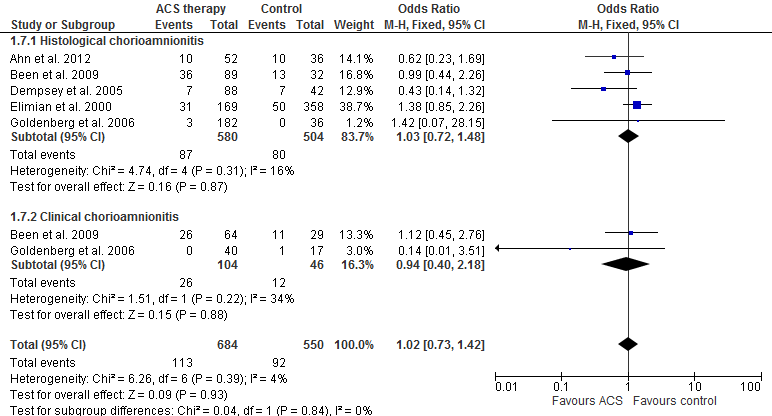


**1.8 Necrotizing enterocolitis (NEC)**


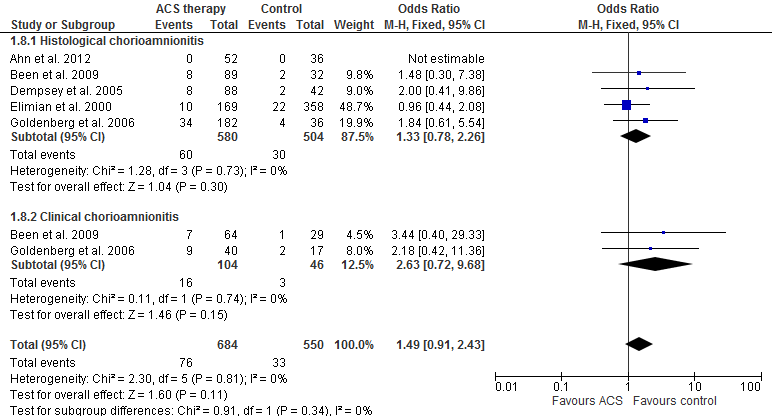


**1.9 Duration of mechanical ventilation, days**


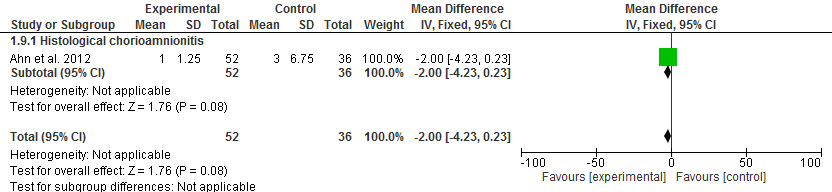


**1.10 Use of mechanical ventilation**


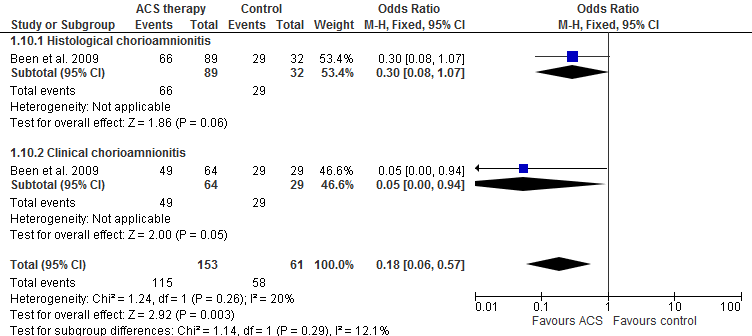


**1.11 Chronic lung disease (CLD) / Bronchopulmonary dysplasia (BPD)**


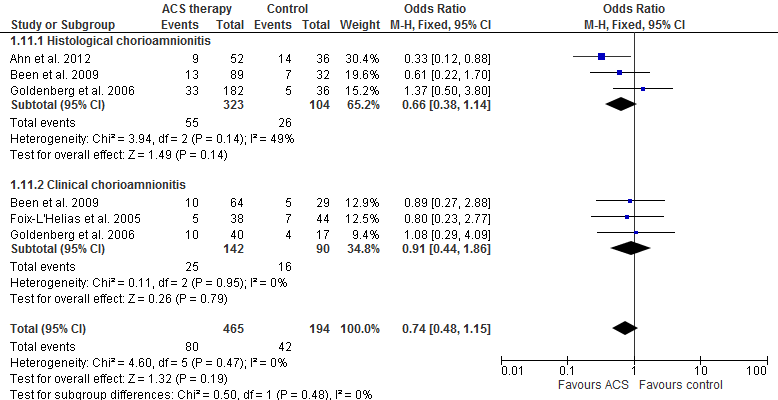


**1.12 Apgar score <7 at 5 min.**


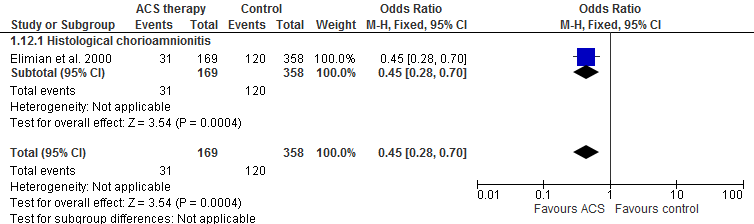


**1.13 Cerebral palsy (at 1 and 3 years’ follow-up)**


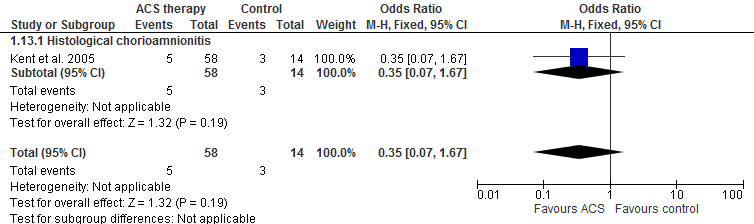


**1.14 General Development Quotient at 1-year follow-up**


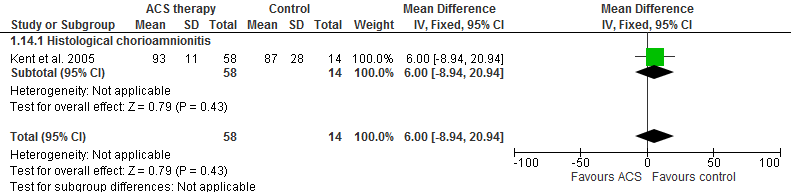


**1.15 General Development Quotient at 3 years’ follow-up**


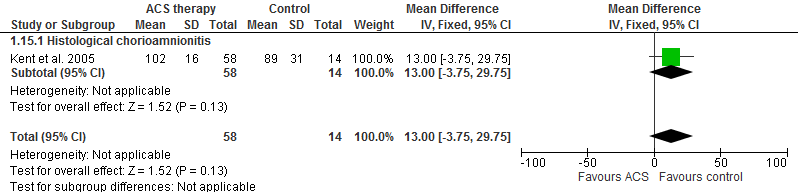

Supplement: S2 File — (DOCX) [file pone.0147604.s002.docx]
