# Supplementary material for: Antenatal Corticosteroids for Reducing Adverse Maternal and Child Outcomes in Special Populations of Women at Risk of Imminent Preterm Birth: A Systematic Review and Meta-Analysis
Source: PLoS One. 2016 Feb 3;11(2):e0147604. doi: 10.1371/journal.pone.0147604 (PMC4740425; doi:10.1371/journal.pone.0147604)
Supplement: S3 File — (DOCX) [file pone.0147604.s003.docx]

**Additional file 3 - Risk of bias assessments for sub-question P3 (women with chorioamnionitis)**

***Risk of bias assessments (Newcastle-Ottawa Scale)***

| **Study ID** | **Ahn 2012** | **Been 2009** | | **Golden-berg 2006** | | **Dempsey 2005** | **Foix-L’Helias 2005** | **Kent 2005** | **Baud 2000** | **Elimian 2000** | |
| --- | --- | --- | --- | --- | --- | --- | --- | --- | --- | --- | --- |
| **SELECTION** | | | | | | | | | | | |
| **Representativeness of exposed cohort**   - Truly representative of the average woman with an ongoing bacterial infection who is at risk of preterm birth (*) - Somewhat representative of the average woman with an ongoing bacterial infection who is at risk of preterm birth (*) - Selected group of users - No description of the derivation of the cohort | * | * | | * | | * | * | * | * | * | |
| **Selection of non-exposed cohort**   - Drawn from the same community as the exposed cohort (*) - Drawn from a different source - No description of the derivation of the non-exposed cohort | * | * | | * | | * | * | * | * | * | |
| **Ascertainment of exposure**   - Secure record (e.g., surgical records) (*) - Structured interview (*) - Written self-report - No description | * | * | | * | | * | * | * | * | * | |
| **Demonstration that outcome of interest not present at study start (i.e., born at institution from which records drawn)**   - Yes (*) - No | * | * | | * | | * | * | * |  | * | |
| **COMPARABILITY^[[1]](#endnote-1)^** | | | | | | | | | | | |
| **Comparability of cohorts on the basis of the design or analysis**   - Study controls for gestational age and/or birth weight (*) - Study controls for any additional factor (*) | (*)  *(not for NEC, PDA, or death)* |  | |  | |  |  |  | *  * |  | |
| **Study ID** | **Ahn 2012** | **Been 2009** | **Golden-berg 2006** | | **Dempsey 2005** | | **Foix-L’Helias 2005** | **Kent 2005** | **Baud 2000** | | **Elimian 2000** |
| **OUTCOME** | | | | | | | | | | | |
| **Assessment of outcome**   - Independent blind assessment (*) - Record linkage (*) - Self report - No description | * | * | | * | | * | * | * | * | * | |
| **Follow-up long enough for outcomes to occur**   - Yes (*) - No | * | * | | * | | * | * | * | * | * | |
| **Adequacy of follow-up of cohorts**   - Complete follow-up – all subjects accounted for (*) - Subjects lost to follow-up unlikely to introduce bias or description provided of those lost (*) - No statement |  | * | |  | | * |  |  |  |  | |
| **Total number of stars** | **7** | **7** | | **6** | | **7** | **6** | **6** | **7** | **6** | |

***Risk of bias assessments (RoBANS)***

| **Study ID** | **Sequence generation** | **Allocation concealment** | **Selection of participants** | **Confounding variables** | **Measurement of exposure** | **Blinding of outcomes assessment** | **Incomplete outcome data** | **Selective outcome reporting** | **Other** |
| --- | --- | --- | --- | --- | --- | --- | --- | --- | --- |
| Ahn 2012  (Pro-spective cohort study) | N/A | N/A | **Low.**  All participants admitted/born at Ewha Womans University during 2005-2010. | **Low/High** (depending on outcome).  Multiple logistic regression models used, controlling for gestational age, at least. However, not used for NEC, PDA, or neonatal death analyses. | **Low**.  Data obtained from direct measurements / clinical assessments. | **Low**.  No statement to indicate blinding, but unlikely to affect outcome measurements. | **Unclear.**  The problem of missing data was deduced from the results; no statement of reason for missing data. | **Low**.  All expected outcomes reported. |  |
| Been 2009  (Pro-spective cohort study) | N/A | N/A | **Low**.  All participants admitted/born at the Erasmus University Medical Center-Sophia Children’s Hospital during May 2001-February 2003. | **High**.  Adjusted analyses not available for separate HCA/CCA results. | **Low**.  Data obtained from direct measurements/ clinical assessments. | **Low**.  No statement to indicate blinding, but unlikely to affect outcome measurements. | **Low**.  No missing data. | **Low**.  All expected outcomes reported. |  |
| Golden-berg 2006  (Retro-spective cohort study) | N/A | N/A | **Low**.  All participants admitted/delivered at same institution during same period (December 5, 1996-June 13, 2001). | **High**.  Adjusted analyses for results stratified by corticosteroid administration not available. | **Low.**  Data obtained from medical records. | **Low.**  No statement to indicate that blinding was performed, but unlikely to affect outcome measurements. | **Unclear**.  No reasons are given for the missing data. | **Low.**  All expected outcomes were reported. |  |
| Dempsey 2005  (Retro-spective cohort study) | N/A | N/A | **Low**.  All participants admitted/delivered at same institution during January 1989-January 1999. | **High**.  Adjusted analyses for results stratified by corticosteroid administration not available. | **Low**.  Data obtained from medical records (obstetrical and neonatal database and pathology database, cross-referenced with data from pathology database and from maternal and neonatal chart review). | **Low**.  No statement to indicate that blinding was performed, but unlikely to affect outcome measurements. | **Unclear**.  No missing data. | **Low**.  All expected outcomes were reported. |  |
| Foix-L'Helias 2005  (Retro-spective cohort study) | N/A | N/A | **Unclear.**  Participants drawn from different institutions, though distribution of treatment and control groups unclear and all during the same time period (1993-1996). | **High.**  Adjusted analyses for results stratified by IUGR not available. | **Low.**  Data obtained from medical records. | **Low.**  No statement to indicate that blinding was performed, but unlikely to affect outcome measurements. | **Unclear.**  No information about missing data. | **Low.**  All pre-defined outcomes reported. | Survey limited to inborn babies, possibly over-estimating the impact of ACT. Yet no distinction made between completed and uncompleted ACT courses, so potential under-estimation. |
| Kent 2005  (Pro-spective cohort study) | N/A | N/A | **Low**.  All participants admitted/delivered at The Canberra Hospital during January 1996-July 2001. | **High**.  Adjusted analyses for results stratified by corticosteroid administration not available. | **Low**.  Data obtained from electronic database. | **Low**.  No statement to indicate that blinding was performed, but unlikely to affect outcome measurements. | **Unclear**.  Low follow-up rate of 53%, though no significant differences for mean birth weight, gestation, exposure to in utero inflammation/infection and cranial US findings between those with and without long-term follow-up data. | **Low**.  All expected outcomes were reported. |  |
| Baud 2000  (Retro-spective cohort study) | N/A | N/A | **Low**.  All participants admitted to Antoine Beclere University Hospital during 1993-1997. | **Low**.  Multiple logistic regression models used, controlling for, antenatal antibiotic administration, mode of delivery, gestational age, and origin (inborn or outborn). | **Low**.  Data obtained from computerized database. | **Low**.  No statement to indicate blinding, but unlikely to affect outcome measurements. | **Unclear**.  Unclear whether incomplete outcome data resulted in low or high risk because the number and reasons for missing data are given without specifying to which group they belong (intervention or control). | **Unclear**. Unclear whether selective outcome reporting resulted in high or low risk. |  |
| Elimian 2000  (Retro-spective cohort study) | N/A | N/A | **Low.**  All participants admitted/delivered at same institution during January 1990-December 1997. | **High**.  Adjusted analyses for results stratified by corticosteroid administration not available. | **Low**.  Data obtained from medical records. | **Low**.  No statement to indicate that blinding was performed, but unlikely to affect outcome measurements. | **Unclear.**  No information about missing data. | **Low**.  All expected outcomes were reported. |  |

1. Adjustments for potential confounding factors, where present, are not incorporated in the meta-analyses. [↑](#endnote-ref-1)
