# Supplementary material for: Antenatal Corticosteroids for Reducing Adverse Maternal and Child Outcomes in Special Populations of Women at Risk of Imminent Preterm Birth: A Systematic Review and Meta-Analysis
Source: PLoS One. 2016 Feb 3;11(2):e0147604. doi: 10.1371/journal.pone.0147604 (PMC4740425; doi:10.1371/journal.pone.0147604)
Supplement: S4 File — (DOCX) [file pone.0147604.s004.docx]

**Additional file 4 – Forest plot for sub-question P4 (women with growth-restricted babies) meta-analyses**

**2.1 Mode of delivery (Caesarean section)**

**
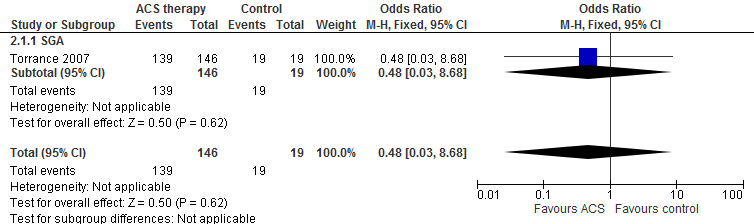
**

**2.2 Chorioamnionitis (histological and/or clinical)**

**
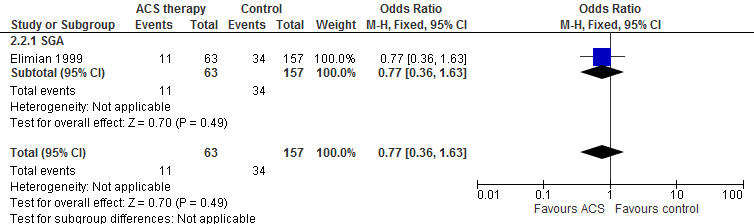
**

**2.3 Perinatal death (fetal or neonatal death)**

**
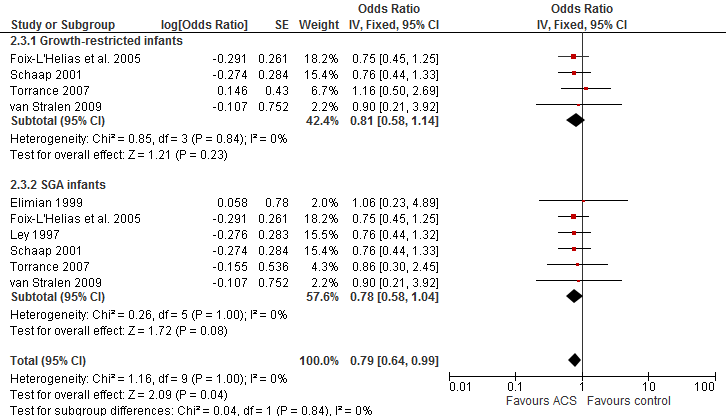
**

*Note*: Generic inverse variance method used for meta-analysis, as one study (Ley 1997) only reports ORs and 95% CIs.

**2.4 Respiratory distress syndrome (RDS)**

**
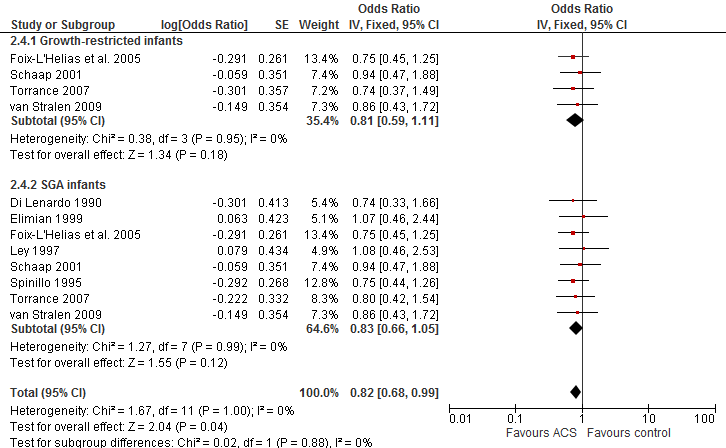
**

Note: Generic inverse variance method used for meta-analysis, as one study (Ley 1997) only reports ORs and 95% CIs.

**2.5 Surfactant use**

**
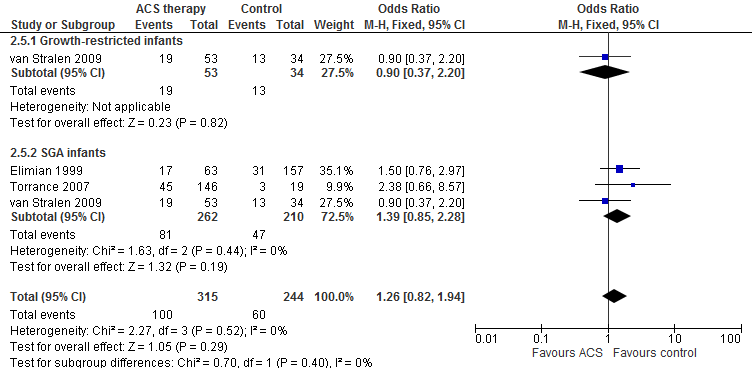
**

**2.6 Major brain lesion (IVH, ICH, PVH, or PVL)**

**
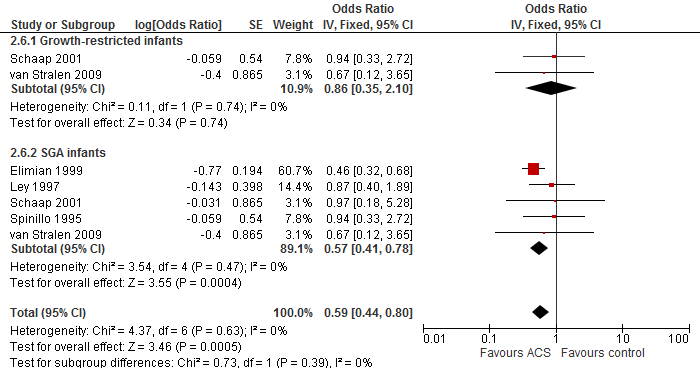
**

Note: Generic inverse variance method used for meta-analysis, as one study (Ley 1997) only reports ORs and 95% CIs.

**2.7 Neonatal sepsis**

**
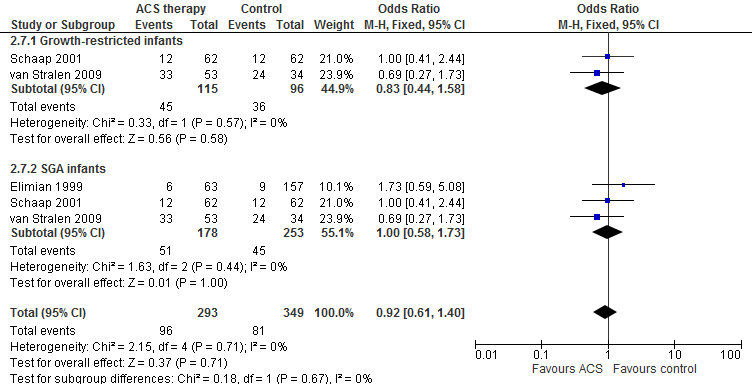
**

**2.8 Necrotizing enterocolitis (NEC)**

**
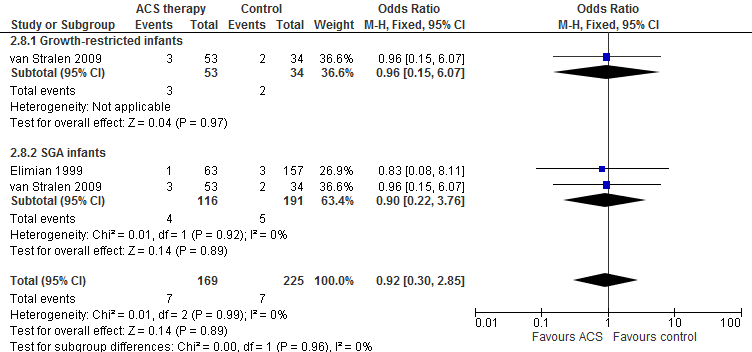
**

**2.9 Chronic lung disease (CLD) / Bronchopulmonary dysplasia (BPD)**

**
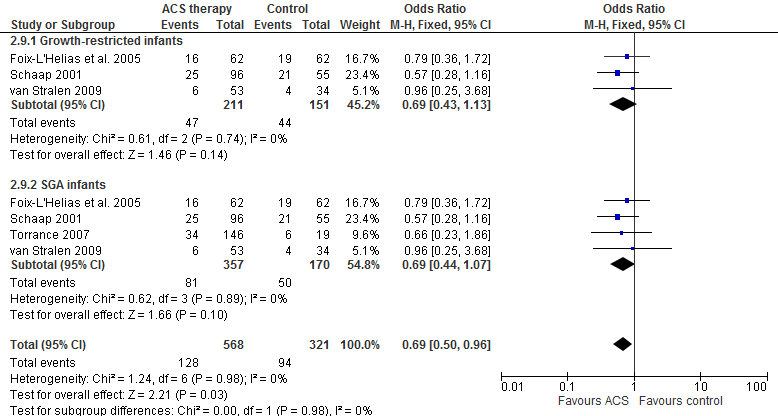
**

**2.10 Patent ductus arteriosus (PDA)**

**
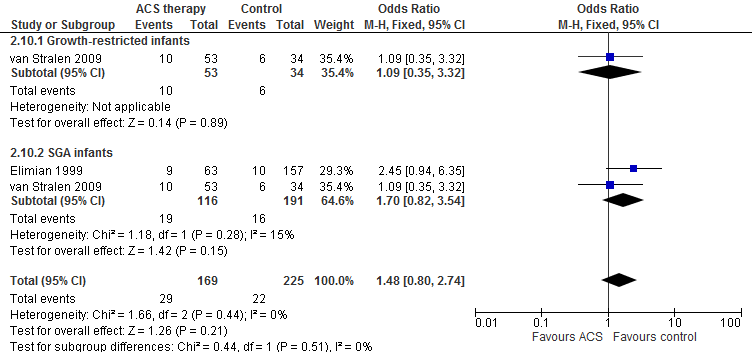
**

**2.11 Low birth weight (<3^rd^ centile for gestational age)**

**
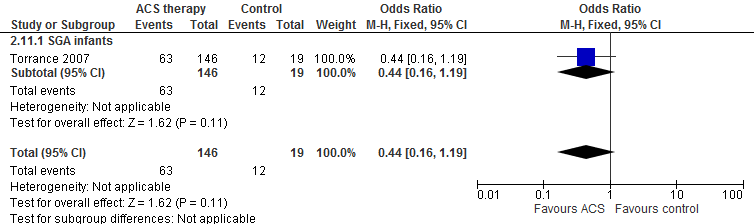
**

**2.12 Duration of mechanical ventilation, days**

**
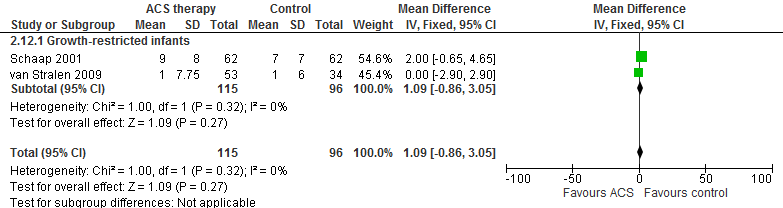
**

**2.13 Use of mechanical ventilation**

**
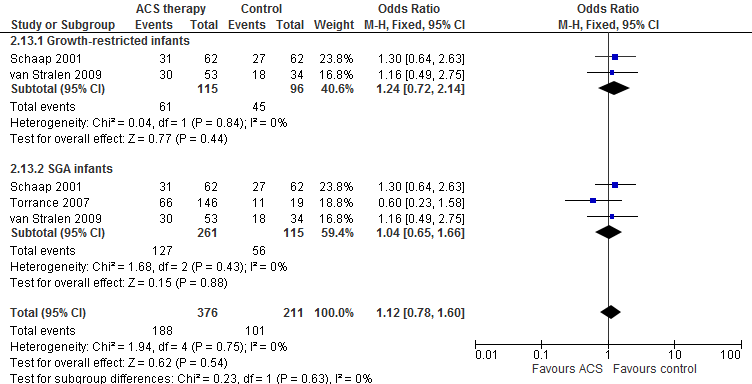
**

**2.14 Apgar score <7 at 5 min.**

**
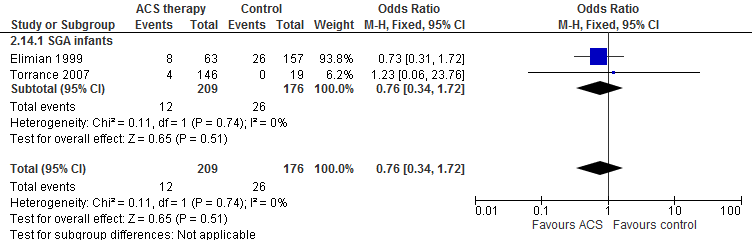
**

**2.15 Growth <10^th^ percentile in early childhood (follow-up to school age)**

**
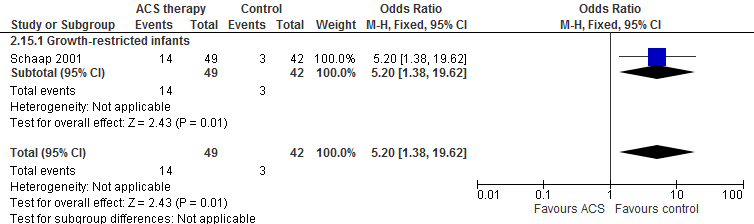
**
