# Supplementary material for: Antenatal Corticosteroids for Reducing Adverse Maternal and Child Outcomes in Special Populations of Women at Risk of Imminent Preterm Birth: A Systematic Review and Meta-Analysis
Source: PLoS One. 2016 Feb 3;11(2):e0147604. doi: 10.1371/journal.pone.0147604 (PMC4740425; doi:10.1371/journal.pone.0147604)
Supplement: S5 File — (DOCX) [file pone.0147604.s005.docx]

**Additional file 5 - Risk of bias assessments for sub-question P4 (women with SGA/growth-restricted babies)**

***Risk of bias assessments (Newcastle-Ottawa Scale)***

| **Study ID** | **van Stralen 2009** | **Torrance 2007** | **Foix-L’Helias 2005** | **Schaap 2001** | **Bernstein 2000** | **Elimian 1999** | **Ley 1997** | **Spinilla 1995** | **Di Lenardo 1990** |
| --- | --- | --- | --- | --- | --- | --- | --- | --- | --- |
| **SELECTION** | | | | | | | | | |
| **Representativeness of exposed cohort**   - Truly representative of the average woman with an IUGR baby at risk of preterm birth (*) - Somewhat representative of the average woman with an IUGR baby at risk of preterm birth (*) - Selected group of users - No description of the derivation of the cohort | * | * | * | * | * | * | * | * | * |
| **Selection of non-exposed cohort**   - Drawn from the same community as the exposed cohort (*) - Drawn from a different source - No description of the derivation of the non-exposed cohort | * | * | * | * | * | * | * | * | * |
| **Ascertainment of exposure**   - Secure record (e.g., surgical records) (*) - Structured interview (*) - Written self-report - No description | * | * | * | * | * | * | * | * | * |
| **Demonstration that outcome of interest not present at study start (i.e., born at institution from which records drawn)**   - Yes (*) - No | * |  | * | * |  | * | * | * | * |
| **COMPARABILITY^[[1]](#endnote-1)^** | | | | | | | | | |
| **Comparability of cohorts on the basis of the design or analysis**   - Study controls for gestational age and/or birth weight (*) - Study controls for any additional factor (*) |  | (*unclear*)  * |  | *  * | *  * |  | (*unclear*)  * | *  * |  |
| **OUTCOME** | | | | | | | | | |
| **Assessment of outcome**   - Independent blind assessment (*) - Record linkage (*) - Self report - No description | * | * | * | * | * | * | * | * | * |
| **Follow-up long enough for outcomes to occur**   - Yes (*) - No | * | * | * | * | * | * | * | * | ***** |
| **Adequacy of follow-up of cohorts**   - Complete follow-up – all subjects accounted for (*) - Subjects lost to follow-up unlikely to introduce bias or description provided of those lost (*) - No statement |  | * |  | * |  |  |  |  |  |
| **Total number of stars** | **6** | **7** | **6** | **9** | **7** | **6** | **7** | **8** | **6** |

***Risk of bias assessments (RoBANS)***

| **Study ID** | **Sequence generation** | **Allocation concealment** | **Selection of participants** | **Confounding variables** | **Measurement of exposure** | **Blinding of outcomes assessment** | **Incomplete outcome data** | **Selective outcome reporting** | **Other** |
| --- | --- | --- | --- | --- | --- | --- | --- | --- | --- |
| van Stralen 2009  (Retro-spective cohort study) | N/A | N/A | **Low.**  All participants admitted/delivered and treated at same institution (Leiden University Medical Center) over same time period (January 2001-December 2005). | **High.**  No confirmation or consideration in either design or analysis phase. | **Low.**  Data obtained from obstetric electronic database. | **Low.**  No statement to indicate that blinding was performed, but unlikely to affect outcome measurements. | **Unclear.**  One child died during emergency caesarean section after eclampsia; unclear how handled. | **Low.**  All pre-defined outcomes reported. | Though equally divided, the difference in origin, i.e. referral pattern, may also have influenced the results. |
| Torrance 2007  (Retro-spective cohort study) | N/A | N/A | **High.**  All participants from single tertiary referral center, admitted to same institution (neonatal intensive care unit at the University Medical Centre Utrecht, the Netherlands) over same period (1 January 1999 - 31 December 2003). Cases and controls thus selected from same pool (e.g., same gestational age, same birth weight). However, control group defined only by no-steroid treatment without further specification, so conceivable that fetal condition on hospitalization differed. Also, absence of outcomes not confirmed at starting point of study as babies delivered elsewhere. | **Low.**  Partial correlation performed for scale data to correct for potential confounding factors; for nominal data, binary logistic regression used for this purpose. Variables were considered to be potential confounders when the Chi-square test or independent t-test identified a significant difference. | **Unclear**.  Data obtained from electronic database, but potential that study outcome of interest present at study start. | **Low.**  No statement to indicate that blinding was performed, but unlikely to affect outcome measurements. | **Low.**  No loss to follow-up. | **Low.**  All pre-defined outcomes reported. |  |
| Foix-L'Helias 2005  (Retro-spective cohort study) | N/A | N/A | **Unclear.**  Participants drawn from different institutions, though distribution of treatment and control groups unclear and all during the same time period (1993-1996). | **High.**  Adjusted analyses for results stratified by IUGR not available. | **Low.**  Data obtained from medical records. | **Low.**  No statement to indicate that blinding was performed, but unlikely to affect outcome measurements. | **Unclear.**  No information about missing data. | **Low.**  All pre-defined outcomes reported. | Survey limited to inborn babies, possibly over-estimating the impact of ACT. Yet no distinction made between completed and uncompleted ACT courses, so potential under-estimation. |
| Schaap 2001  (Case-control study) | N/A | N/A | **Unclear.**  Participants drawn from different institutions, though distribution of treatment and control groups unclear and all during the same time period (1984-1991). Possibility of selection bias cannot be excluded due to retrospective design. | **Low.**  Treated group matched with control group by random electronic selection based on birth weight (difference < 175 g), sex, and year of birth (difference less than 2 years). | **Low.**  Data obtained from medical records. Because all mothers had been admitted at least 24 hrs before delivery, a difference in fetal condition on admission was unlikely. | **Low.**  No statement to indicate that blinding was performed, but unlikely to affect outcome measurements. | **Low.**  Nine losses at school age follow-up (4 in steroid group, 5 in control group) but no significant difference in socio-demographic details between those lost and retained at follow-up. | **Low.**  All pre-defined outcomes reported. | Hypertensive mothers less often treated with corticosteroids. Also, matching notwithstanding, birth weight and gestational age were significantly lower in the AG group, though magnitude of difference small. |
| Bernstein 2000  (Retro-spective cohort study) | N/A | N/A | **High.**  Participants drawn from different institutions, though distribution of treatment and control groups unclear and all during the same period (1991-1996). Also, absence of outcomes not confirmed at starting point of study as babies delivered elsewhere. | **Low.**  Following potential confounders controlled for: gestational age, race, route of delivery, use of prenatal care, gender, and location of birth (birth within a network institution vs. postnatal transfer to a network institution). Stepwise logistic regression performed. | **Unclear**.  Data obtained from electronic database, but potential that study outcome of interest present at study start. | **Low.**  No statement to indicate that blinding was performed, but unlikely to affect outcome measurements. | **Unclear.**  No information about missing data. | **Low**.  All pre-defined outcomes reported. |  |
| Elimian 1999  (Retro-spective cohort study) | N/A | N/A | **Unclear.**  All participants from same institution during same period (January 1990-July 1997), but control group defined only by no-steroid treatment without further specification, so conceivable that fetal condition on hospitalization differed. | **High.**  Consideration in design but no adjusted stratified analysis for sub-sample of interest. | **Low.**  Data obtained from medical records. | **Low.**  No statement to indicate that blinding was performed, but unlikely to affect outcome measurements. | **Unclear**.  No information about missing data. | **Low.**  All pre-defined outcomes reported. |  |
| Ley 1997  (Retro-spective cohort study) | N/A | N/A | **Low.**  All participants admitted/delivered and treated at same institution (University Hospital of Lund) during same period (1985-1994). | **Unclear.**  Multiple logistic regression performed, but inclusion of confounding factors not specified. | **Low.**  Data obtained from hospital records. | **Low.**  No statement to indicate that blinding was performed, but unlikely to affect outcome measurements. | **Unclear.**  No information about missing data. | **Low.**  All pre-defined outcomes reported. |  |
| Spinillo 1995  (Pro-spective cohort study) | N/A | N/A | **Unclear.**  All participants from same institution during same period (1988-1993), but control group defined only by no-steroid treatment without further specification, so conceivable that fetal condition on hospitalization differed. | **Low.**  Multivariate models used to account for potential confounders. (age, birth weight, and sex of the infant) | **Low.**  Data obtained from hospital records. | **Low.**  No statement to indicate that blinding was performed, but unlikely to affect outcome measurements. | **Unclear.**  No information about missing data. | **Low.**  All pre-defined outcomes reported. |  |
| Di Lenardo 1990  (Retro-spective cohort study) | N/A | N/A | **Unclear.**  All participants admitted/delivered and treated at same institution (Prenatal Care Ward of Univ. of Padua’s Gynaecology & Obstetrics Institution), but unclear whether over same period. | **High.**  No confirmation or consideration in either design or analysis phase. | **Low.**  Data obtained from medical records. | **Low.**  No statement to indicate that blinding was performed, but unlikely to affect outcome measurements. | **Unclear.**  No information about missing data. | **Low.**  All pre-defined outcomes reported. |  |

1. Adjustments for potential confounding factors, where present, are not incorporated in the meta-analyses. [↑](#endnote-ref-1)
